# Lactotransferrin upregulation affects the pathological changes of non-small cell lung cancer by regulating ferroptosis

**DOI:** 10.7717/peerj.20866

**Published:** 2026-02-27

**Authors:** Yuxiu Wang, Wenjing Xu, Kaiqi Ren, Lingfeng Min

**Affiliations:** Department of Respiratory and Critical Care Medicine, Northern Jiangsu People’s Hospital Affiliated to Yangzhou University, Yangzhou, Jiangsu, China

**Keywords:** Non-small cell lung cancer (NSCLC), Lactotransferrin (LTF), Ferroptosis, Epithelial mesenchymal transformation (EMT)

## Abstract

**Background:**

Recent studies have highlighted the role of ferroptosis, a form of regulated cell death driven by iron-dependent lipid peroxidation, in cancer biology. This study aims to investigate the effect of lactotransferrin (LTF) upregulation on the pathological changes related to non-small cell lung cancer (NSCLC) *via* the inhibition of ferroptosis.

**Methods:**

LTF’s involvement in NSCLC was investigated through cell experiments and clinical samples. Cell models with stable LTF knockdown or overexpression were established by lentiviral transduction. Cell viability and cytotoxicity were evaluated through cell counting kit 8 (CCK8) and lactate dehydrogenase (LDH) experiments. Scratch and Transwell experiments were conducted to verify the effect of LTF expression on the migration and invasion abilities of lung cancer cells. Protein and mRNA expression were analyzed using Western blotting and qPCR. Malondialdehyde (MDA), glutathione (GSH), free iron ions (Fe^2+^), and reactive oxygen species (ROS) levels were measured with appropriate kits. The intracellular localization and expression of the protein was detected through immunofluorescence (IF). Peripheral blood of healthy controls and patients with preliminarily diagnosed non-small cell lung cancer was collected, and the expression levels of LTF protein and mRNA were detected by Western blotting and quantitative polymerase chain reaction (qPCR) experiments.

**Results:**

The results demonstrate that LTF was upregulated in NSCLC and it’s overexpression could significantly enhance the migration, invasion, and epithelial-mesenchymal transition (EMT) of non-small cell lung cancer cells. The overexpression of LTF significantly inhibited ferroptosis in non-small cell lung cancer cells. LTF modulates the expression of critical regulators of ferroptosis including glutathione peroxidase 4 (GPX4) and acyl-CoA synthetase long-chain family member 4 (ACSL4), leading to altered cellular redox status. The protein and mRNA expression levels of LTF were both increased in the peripheral blood of patients with NSCLC, with changes in protein level being more significant. Additionally, the overexpression of LTF was significantly correlated with the stage of NSCLC.

**Conclusion:**

In conclusion, these findings suggest that LTF upregulation plays a crucial role in inhibiting ferroptosis, thereby influencing the pathological progression of NSCLC. This study provides a potential therapeutic avenue for targeting ferroptosis in NSCLC treatment strategies.

## Introduction

Non-small cell lung cancer (NSCLC) is one of the most prevalent forms of lung cancer, accounting for approximately 85% of all lung cancer cases worldwide (Zhang et al., 2023). The incidence of NSCLC continues to rise, with an estimated 2.2 million new cases and 1.8 million deaths reported globally in 2020, making it a significant public health concern (Li et al., 2023). The aggressive nature of NSCLC, coupled with its late-stage diagnosis, often results in poor prognoses and limited treatment options. Current clinical treatments, including surgery, chemotherapy, and targeted therapies, have shown varying degrees of efficacy. However, resistance to these treatments frequently develops, leading to disease recurrence and metastasis (Liu et al., 2024). Furthermore, the heterogeneity of NSCLC complicates treatment strategies, as different subtypes may respond differently to standard therapies. Recent research has highlighted the role of ferroptosis, a form of regulated cell death characterized by iron-dependent lipid peroxidation, in cancer progression and treatment resistance (Li et al., 2024b). Understanding the molecular mechanisms underlying ferroptosis in NSCLC could provide new insights into potential therapeutic targets and strategies to improve patient outcomes.

Lactotransferrin (LTF) is a multifunctional glycoprotein primarily known for its role in iron transport and homeostasis. It binds iron with high affinity, thereby regulating its availability in biological systems; this is crucial for various cellular processes, including cell proliferation and differentiation (Leveugle et al., 1994). Emerging evidence suggests that LTF plays a significant role in modulating ferroptosis, a form of regulated cell death characterized by the accumulation of lipid peroxides and iron dependency (Wang et al., 2020). Mechanistically, LTF sequesters free iron ions (Fe^2+^/Fe^3+^), thereby limiting iron-catalyzed lipid peroxidation—a hallmark of ferroptosis (Wang et al., 2024). Beyond its iron-binding capacity, LTF is involved in other biological functions including antimicrobial activity, immunomodulation, and antioxidation. Recent studies indicate that LTF suppresses pro-inflammatory pathways (*e.g*., NF-κB/COX-2; Yami et al., 2023; Habib et al., 2023), which are known to exacerbate ferroptosis under certain conditions (Yao et al., 2021; Li et al., 2021). However, the precise molecular targets of LTF in ferroptosis regulation are not yet fully understood. This study aims to elucidate the specific mechanisms by which LTF influences ferroptosis, focusing on iron metabolism and redox balance.

LTF dysregulation has been implicated in promoting tumor progression and metastasis in NSCLC (Iijima et al., 2006; Wen et al., 2023), as it may lead to altered iron metabolism, contributing to the oxidative stress environment that favors cancer cell survival and proliferation (Golabek et al., 2022; Liu et al., 2022; Zhang et al., 2022; Zhao, Cheng & Xiong, 2021). Furthermore, the interplay between LTF and ferroptosis in NSCLC remains an area of active investigation, with emerging evidence suggesting that LTF may modulate the sensitivity of cancer cells to ferroptosis stimuli (Wang et al., 2020, 2024). However, the precise molecular mechanisms underlying LTF’s role in NSCLC and their potential as therapeutic targets are still not fully understood, highlighting a significant gap in current research. Further elucidation of these pathways could provide valuable insights into the development of novel therapeutic strategies for NSCLC.

This study aims to investigate the role of LTF upregulation in the pathological changes associated with NSCLC through the modulation of ferroptosis. Elucidating the relationship between LTF levels and ferroptosis in NSCLC could provide insight into the underlying mechanisms that contribute to tumor progression and resistance to therapy. The significance of this research lies in its potential to identify novel therapeutic targets and strategies for enhancing the efficacy of existing treatments for NSCLC. Understanding how LTF influences ferroptosis could pave the way for innovative approaches to manipulate cancer cell survival and promote tumor regression. Ultimately, this study aims to contribute to the growing body of knowledge regarding the interplay between iron metabolism and cancer biology, with the hope of improving clinical outcomes for patients suffering from NSCLC.

## Methods

### Reagents

Ferrostain-1 (HY-100579) was purchased from MedChemExpress (Shanghai, China).

### Analysis of public data sets of LTF in normal and NSCLC lung tissues

The RNA-sequencing-based gene expression data of 1,041 NSCLC tumor samples and 108 para-cancer samples were downloaded from The Cancer Genome Atlas (TCGA, https://portal.gdc.cancer.gov/). The public datasets of GSE18842, GSE19188, GSE40791, and GSE116959 were used (https://www.ncbi.nlm.nih.gov/gds/). TCGA data were normalized to Fragments Per Kilobase Million (FPKM), and Gene Expression Omnibus (GEO) datasets were converted to TPM for cross-study comparability. The expression differences of LTF between NSCLC and para-cancer samples were confirmed using “ggplot2[3.4.4]” packages in R (4.2.1; R Core Team, 2022), with thresholds set at |log2FC| > 1 and adjusted *P*-value (FDR) < 0.05. Normalization methods and thresholds were consistent with previous study (Xiao et al., 2021).

The distribution of LTF protein expression was determined by immunohistochemistry staining based on the Human Protein Atlas (THPA) (https://www.proteinatlas.org/).

### Cell lines

The cell lines A549 and H1299 were acquired from the Shanghai Institute of Life Sciences. The cells were cultured under controlled conditions in a 1,640 constant temperature incubator, supplemented with 10% fetal bovine serum, at a temperature of 37 °C and an atmosphere of 5% CO2.

### Lentivirus transduction and generation of stable cell lines

The cell lines A549 and H1299 were cultured and reached a confluence of around 30–50%. The prepared LTF-overexpressing lentiviral particles and LTF-silencing lentiviral particles (Kaiji Gene, Jiangsu, China) were added to the target cell culture medium together with LV-Enhance (with a final concentration of approximately 4–8 μg/mL). The specific details of the construction of the lentiviral vector, including the sequences, backbone vector name, multiplicity of infection (MOI), and validation efficiency images are elaborated in File S2. The cells were incubated at 37 °C and 5% CO_2_ for 12–24 h. After this incubation period, the medium was replaced with fresh complete medium. Then, the selection process was initiated by adding puromycin to the medium. The cells were continuously cultured with the puromycin, and the medium was replaced every 2–3 days. After several weeks of selection, the surviving colonies were expanded, and stable integration of the target gene was confirmed by genomic PCR, and its expression was verified at both transcriptional (qPCR) and protein levels (Western blot).

### Cell migration assay

First, cells were inoculated in the logarithmic growth phase into a 6-well plate. Once the cells were fully adherent and reached a confluence of 80–90%, a sterile 20 μL pipette tip was used to make a straight scratch vertically and evenly across the cell monolayer to create a cell free scratch area. Then, the cells were gently washed three times with phosphate-buffered saline (PBS) to remove detached cell debris, and serum-free medium containing different treatment factors (ferrostain-1) was added. Next, the 6-well plate was placed in a cell incubator at 37 °C with 5% CO_2_. At 0, 12, 24, and 48 h, pictures were taken of fixed fields of view under an inverted microscope to record the changes in the scratch area. Finally, the scratch width was measured at different time points using ImageJ software, and the cell migration rate was calculated to evaluate the strength of cell migration ability.

### Transwell

The transwell inserts were rehydrated by immersing them in the appropriate culture medium within a 24-well plate for 1.5 h, allowing for environmental equilibration. Subsequently, cells in the logarithmic growth phase were harvested and enzymatically dissociated into a single-cell suspension, and the cell density was then adjusted accordingly. Prior to the addition of cells, the membrane of the upper chamber was coated with Matrigel. A complete medium was introduced in the lower chamber. The plate was then incubated under standard conditions for a period of 24 h. After incubation, the non-invaded cells were carefully removed from the upper surface of the membrane with a cotton swab. The cells that had migrated or invaded to the lower surface of the membrane were fixed using a fixative for 20 min, after which they were stained with crystal violet for 15 min. Finally, the stained cells were observed and counted under a microscope to quantify the cell invasion ability.

### Cell viability assay (CCK8)

Cell proliferation was assessed using a commercial CCK-8 kit (Beyotime, Shanghai, China). Cells were briefly seeded in 96-well plates at a density of 5 × 10^3^ cells/well in 200 μl culture medium. Following 24-h incubation, 10 μl of CCK-8 reagent was added to each well, followed by an additional 4-h incubation. Optical density was then measured at 450 nm using a microplate reader (Tecan, Mannedorf, Switzerland).

### Cytotoxicity assay (LDH)

Cytotoxicity was measured using a lactate dehydrogenase (LDH) release assay (MedChemExpress, Shanghai, China). Cells were seeded in 96-well plates (10,000 cells/well), including blank control wells (medium only), negative control wells (untreated cells), and experimental group wells. After treatment, supernatants were transferred to a fresh plate, mixed with LDH substrate, and incubated in the dark (30 min). Absorbance at 490 nm was recorded, with cytotoxicity calculated relative to controls.

### Intracellular reactive-oxygen species (ROS) detection

Intracellular reactive oxygen species (ROS) levels were measured using a DCFH-DA fluorescent probe (Nanjing Jincheng Bioengineering Institute, Jiangsu, China). Cells cultured in 6-well plates were incubated with DCFH-DA (diluted 1:1,000) for 30 min at 37 °C in the dark. After three PBS washes, ROS-associated fluorescence was visualized using a laser scanning confocal microscope (Nikon Instruments Inc, Tokyo, Japan).

### Malondialdehyde and glutathione detection

Intracellular malondialdehyde (MDA) levels were measured using a commercial MDA assay kit (Nanjing Jiancheng Bioengineering Institute, Jiangsu, China). The sample was heated with thiobarbituric acid (TBA) at 95 °C for 40 min under acidic conditions to form a pink MDA-TBA adduct. Absorbance was read at 532 nm, and MDA concentrations were calculated using a standard curve, expressed as nmol per mg protein (nmol/mg protein).

Glutathione (GSH) levels were quantified using a commercial GSH assay kit (Nanjing Jiancheng Bioengineering Institute, Jiangsu, China). Cell lysates were deproteinized with 5% sulfosalicylic acid (SSA) on ice for 10 min, followed by centrifugation at 12,000g to remove protein-bound thiols. The supernatant was mixed with 0.1 M phosphate buffer (pH 8.0) and 1 mM DTNB, incubated for 10 min at room temperature to generate a yellow-colored product. Absorbance was measured at 412 nm, and GSH concentrations were calculated based on a standard curve, normalized to total protein content (μmol/mg protein).

### Measurement of intracellular Fe^2+^

Intracellular Fe^2+^ levels were determined using a commercial ferrous iron fluorometric assay kit (Wuhan, China). Cells were seeded on coverslips for 24 h, then the cell culture medium was removed and cells were washed twice with PBS. The 10 mmol ferrous iron fluorometric probe was added, and cells were incubated for 1 h at 37 °C in the dark. Following three buffer washes, fluorescence was visualized by Nikon A1Si confocal microscopy (Nikon Instruments Inc., Tokyo, Japan).

### Immunofluorescence

Cell slide samples were prepared using 24-well cell culture plates and fixed by 4% paraformaldehyde to preserve structure and antigenicity. Next, the non-specific binding sites on the sample were blocked with BSA. The primary antibody was then added for 4 °C overnight. The sample was thoroughly washed with TBST to remove unbound primary antibodies. The fluorescently-labeled secondary antibody that targets the primary antibody was then added, and the sample was incubated again for 1 h in room temperature. The sample was washed once more to eliminate unbound secondary antibody, and the nuclei were then stained with DAPI for 15 min. Finally, the sample was mounted with a fluorescence-compatible mounting medium and observed under a fluorescence microscope. The following primary antibodies were used: E-cadherin (Santa, mouse, 1:200) and N-cadherin (Santa, mouse, 1:200). The following fluorescently-labeled secondary antibodies were used: FITC-labeled Goat Anti-Mouse IgG (H+L) and Cy3-labeled Goat Anti-Mouse IgG (H+L).

### Western blot

RIPA lysis buffer (Beyotime, Shanghai, China) was used to extract total protein from the cells. The total protein concentration was determined using the bicinchoninic acid (BCA) assay (Vazyme, Nanjing, China). A total of 40 μg protein was separated *via* 10% SDS-PAGE and subsequently transferred to polyvinylidene difluoride (PVDF) membranes. Following a blocking step with 5% bovine serum albumin, the membrane was incubated overnight at 4 °C with the appropriate primary antibody. After washing, the membrane was incubated for 1 h at room temperature with a secondary antibody (HRP conjugated Affinipure Goat anti-mouse antibody, SA00001-1 or HRP conjugated Affinipure Goat anti-rabbit antibody, SA00001-2; ProteinTech, Rosemont, IL, USA), followed by detection using an enhanced chemiluminescence kit. Western blotting was conducted using monoclonal mouse anti-LTF antibody (1:500; Abcam, Cambridge, UK), monoclonal mouse anti-E-cadherin (1:500; Santa, New Delhi, India), monoclonal mouse anti-N-cadherin (1:500; Santa, New Delhi, India), monoclonal mouse anti- ACSL4 (1:2,000; ProteinTech, Rosemont, Illinois, USA), monoclonal ribbit anti- GPX4 (1:2,000; Abmart, Berkeley Heights, NJ, USA), monoclonal ribbit anti-Tubulin-β (1:3,000; Beyotime, Shanghai, China), and monoclonal mouse anti-GAPDH antibody (1:5,000; ProteinTech, Rosemont, IL, USA). The resulting images were captured using the Gel Dox XR system (Bio-Rad, Hercules, CA, USA).

### Transmission electron microscopy

The cells specimens were fixed with electron microscope fixative to preserve their structures (Servicebio, Wuhan, China) and were transported at 4 °C. Wuhan Servicebio Biological Company was entrusted to assist in this process.

### Patients

A total of 48 peripheral blood samples comprising 12 normal and 36 NSCLC patients were collected at the Biobank of Northern Jiangsu People’s Hospital during the period from July 2024 to December 2024. Written informed consent was obtained from all patients, and the study protocols concerning the use of human samples were approved by the Ethics Committee of Northern Jiangsu People’s Hospital, with the approval number: 2021KY-053.

### Enzyme-linked immunosorbent assay (ELISA)

An LTF ELISA assay kit (Wuhan Huamei Bioengineering Company, China) was used to detect plasma LTF levels. First, a plasma sample was added to a 96-well enzymic plate coated with LTF antibody for 1 h at 37 °C. After washing away unbound impurities, an enzyme-labeled specific antibody was added for 30 min at room temperature; this formed an immune complex with the target substance already bound to the solid-phase carrier. After another round of washing, the enzyme substrate was added for 30 min at room temperature. Under the catalytic action of the enzyme, the substrate undergoes a color-development reaction. Finally, the absorbance value was measured by a microplate reader, and the concentration of the target substance in the sample was calculated based on the standard curve.

### RNA extraction from peripheral blood samples

Fresh ethylenediaminetetraacetic acid (EDTA) anticoagulated human peripheral blood (5 mL) was collected from eight normal and 6 NSCLC patients. Red blood cells were removed through red blood cell lysis buffer (lysed at 4 °C for 10 min, centrifuged at 1,500×g, and supernatant discarded), and white blood cell precipitates were collected. An RNAprep Pure Blood Kit (TIANGEN) was then used to extract peripheral blood RNA. A 350 μL RB buffer + 20 μL Proteinase K was mixed with the pellet, vortexed thoroughly, and incubated at 56 °C for 10 min. The lysate was loaded onto the CR3 spin column and centrifuge at 12,000×g for 30 s, and the flow-through was then discarded. Next, a 500 μL RW1 buffer was added (centrifuged for 30 s), followed by a 500 μL RW2 buffer (centrifuged for 1 min). The column was dried by centrifugation at 12,000×g for 2 min. It was then transferred to a new tube, mixed with 30 μL RNase-free water, incubated for 1 min, and centrifuged at 12,000×g for 1 min to collect RNA. The RNA concentration was determined by ultraviolet spectrophotometry (NanoDrop; absorbance at 260 nanometers).

### Quantitative real-time polymerase chain reaction (RT-qPCR)

A PrimeScriptTM RT kit (Takara Bio, Kusatsu, Shiga, Japan) was used to perform reverse transcription. Quantitative PCR was carried out using Takara’s SYBR Green PCR Master Mix on the StepOnePlus system (Takara Bio, Kusatsu, Shiga, Japan). Ploidy changes at the gene level were determined using the 2^−∆∆CT^ method, with GAPDH as the normalization gene. The primer sequences for qRT-PCR were as follows: LTF-Forward: 5′-CCGCCGTGGACAGGACTG-3′; LTF-Reverse: 5′-CGCCAATACACAGAGCACAGAG-3′; GAPDH-Forward: 5′-GGAAGCTTGTCATCAATGGAAATC-3′; GAPDH-Reverse: 5′-TGATGACCCTTTTGGCTCCC-3′.

The discrepancy in sample sizes between ELISA (*n* = 48) and RT-qPCR (*n* = 14) assays was due to insufficient cDNA quantities derived from a subset of blood specimens during RNA extraction. To ensure data reliability, samples with low RNA quality or concentration were excluded from RT-qPCR analysis. All included samples passed rigorous quality controls (*e.g*., A260/A280 ratio ≥1.8, RNA integrity number >7).

### Statistical analysis

Prism 9.0.1 (GraphPad, La Jolla, CA, USA) was used for data analysis. All data are presented as mean values with standard deviation (SD). The two-tailed Student’s t-test was performed to compare the differences between two groups. Analysis of variance (ANOVA) was used to analyze the differences among multiple sets of data. The Chi-square test was used to analyze the differences among categorical variable and the Mann-Whitney U test was used to analyze the differences among categorical variable. ImageJ software was used to detect the gray value of protein expression. All *in vitro* experiments were repeated three times. Data were considered statistically significant when the *P*-value was less than 0.05.

## Results

### LTF was overexpressed in NSCLC

**Figure 1:**
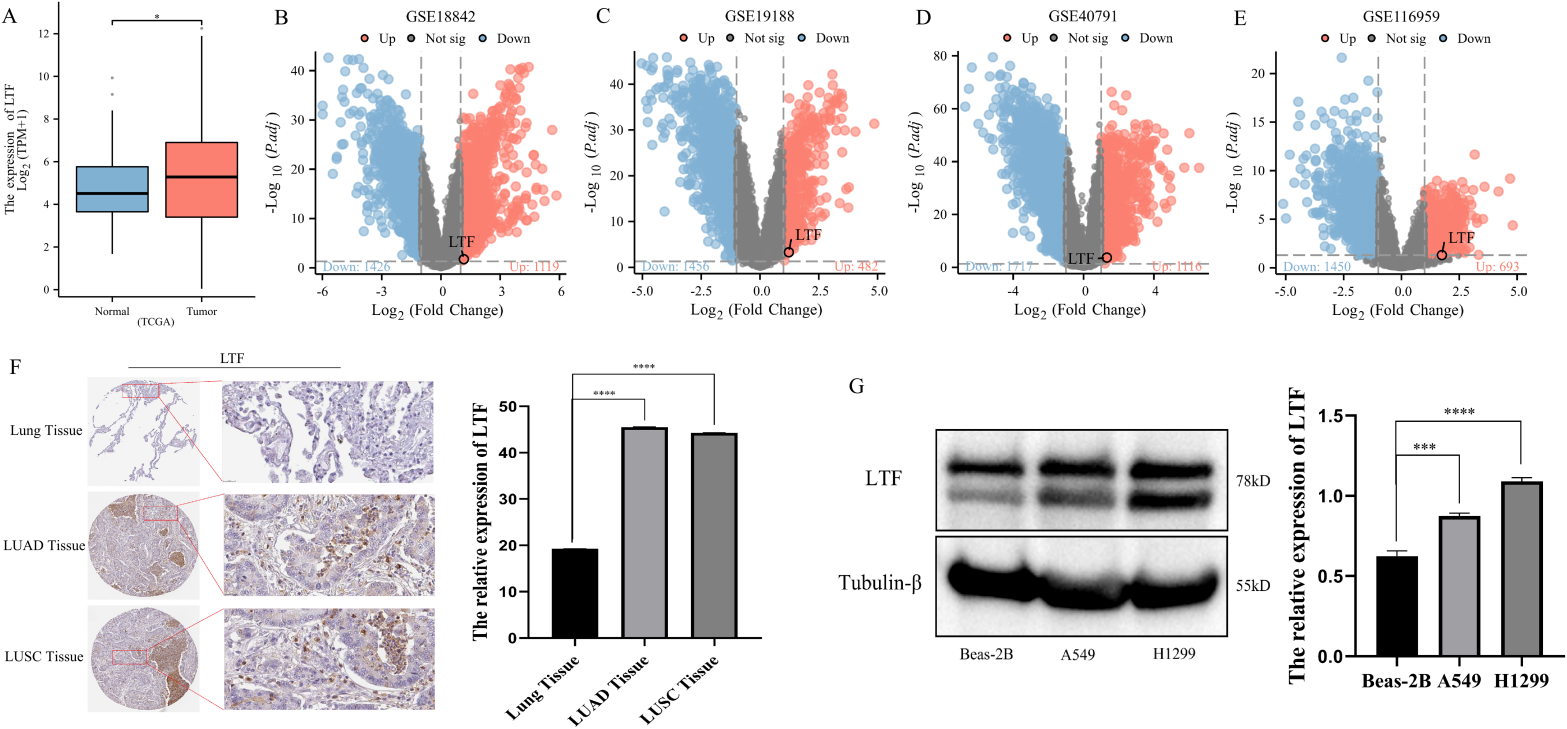
The expression of LTF in NSCLC. (A) Volcano plots showing differential expressed genes (DEGs) of NSCLC in the TCGA group dataset. (B–E) Volcano plots showing differential expressed genes (DEGs) of NSCLC in the GSE18842, GSE19188, GSE40791 and GSE116959 dataset. (F) Immunohistochemistry from THPA database data showing different expression levels of LTF in NSCLC tissues. (G) Western blotting showing different expression levels of LTF in Beas-2B, A549 and H1299 cells. (*n* = 3. The values are expressed as the means ± SD. *P < 0.05, ***P < 0.001 and ****P < 0.0001.)

The alteration of LTF levels was detected in lung cancer tissues and cell lines. LTF mRNA levels were highly expressed in lung cancer, as observed by combining TCGA and GEO data analyses (Figs. 1A–1E). LTF was also significantly upregulated in both lung adenocarcinoma (LADC) and lung squamous cell carcinoma (LUSC) from The Human Protein Atlas (THPA) database (Fig. 1F). The expression level of LTF was significantly increased in NSCLC cells (Fig. 1G).

### LTF knockdown can inhibit the migration and invasion of NSCLC cells

**Figure 2:**
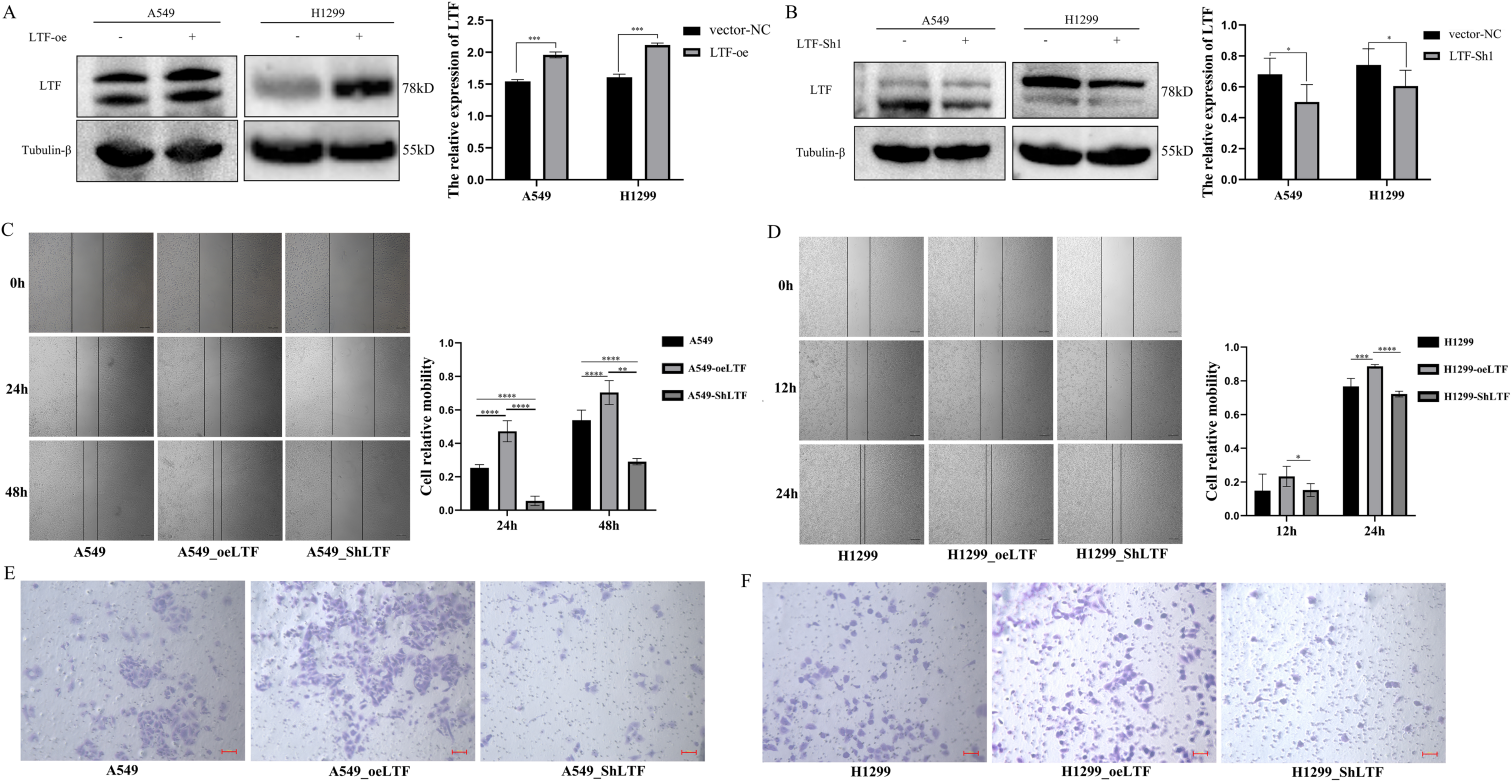
The expression of LTF affects the migration and invasion of NSCLC cells. (A, B) Silenced and overexpressed LTF in A549 and H1299 cells. (C, D) Cell migration experiment showing different cells migration rate of silenced and overexpressed LTF cells. (E, F) Transwell experiment showing different infiltration of silenced and overexpressed LTF cells. (*n* = 3. The values are expressed as the means ± SD. *P < 0.05, **P < 0.01, ***P < 0.001 and ****P < 0.0001.)

To clarify the role of LTF in the pathogenesis of NSCLC, LTF was silenced and overexpressed in A549 and H1299 cells (Figs. 2A, 2B). The results of cell scratch experiments showed that overexpression of LTF significantly increased the A549 and H1299 cell migration rate, while silencing LTF significantly decreased the A549 and H1299 cell migration rate; these difference was statistically significant (*P* < 0.05; Figs. 2C, 2D). In addition, the results of the Transwell experiments showed that overexpression of LTF significantly increased the infiltration of A549 and H1299 cells, and silencing LTF significantly decreased the infiltration of A549 and H1299 cells (Figs. 2E, 2F).

### LTF knockdown can inhibit EMT of NSCLC cells

**Figure 3:**
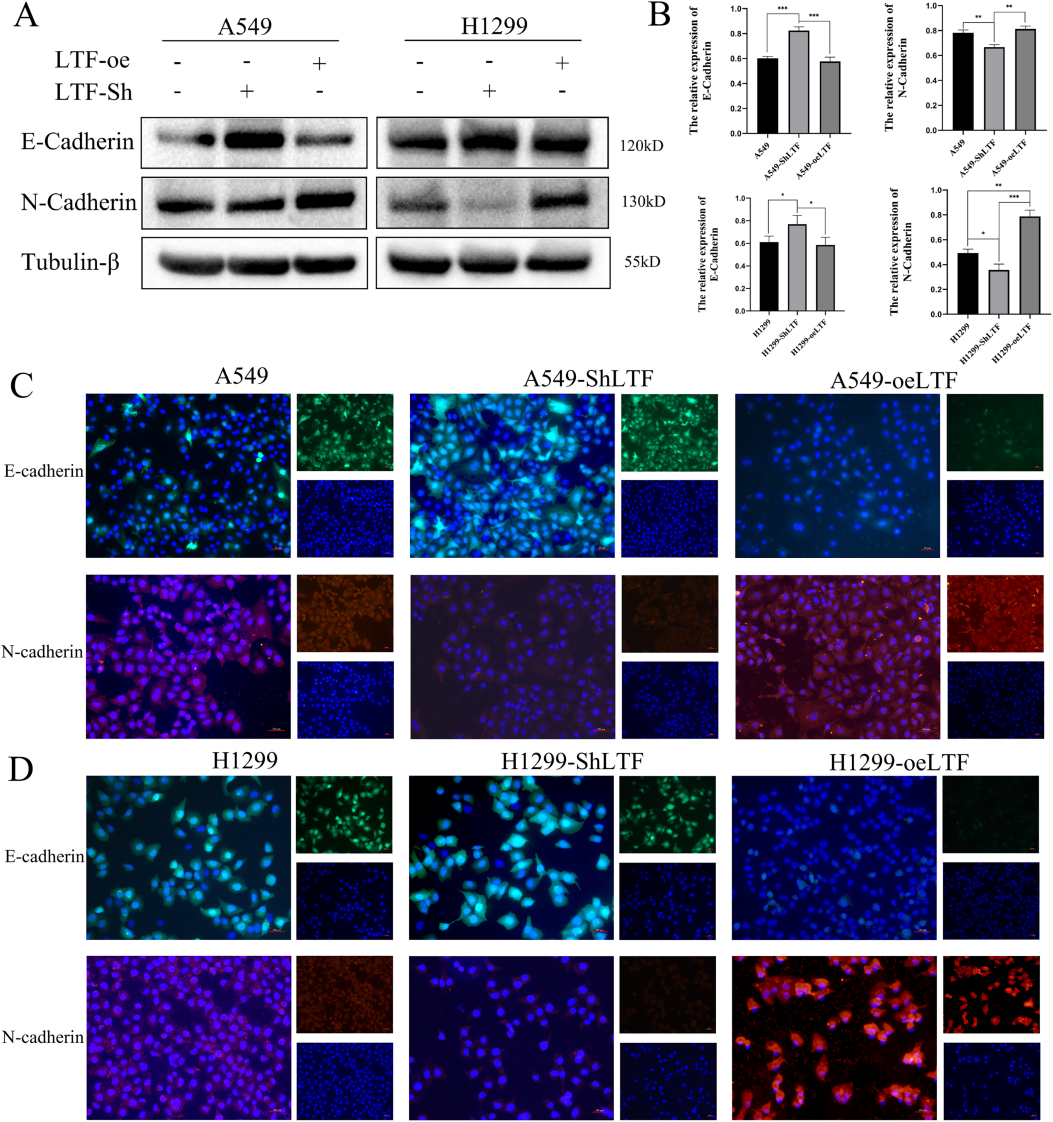
The expression of LTF affects the EMT of NSCLC cells. (A, B) Western blotting showing different expression levels of E-cadherin and N-cadherin in silenced and overexpressed LTF cells. (C, D) Immunofluorescence staining showing different expression levels of E-cadherin and N-cadherin in silenced and overexpressed LTF cells. (*n* = 3. The values are expressed as the means ± SD. *P < 0.05, **P < 0.01 and ***P < 0.001.)

It is well known that epithelial-mesenchymal transition (EMT) changes are significant to tumor pathology. EMT changes in A549 cells and H1299 cells were analyzed during LTF silencing and overexpression. The results of western blot and immunofluorescence experiments showed that epithelial marker (E-cadherin) expression was significantly up-regulated while interstitial marker (N-cadherin) expression was significantly down-regulated after LTF knockdown, and epithelial marker (E-cadherin) expression was significantly down-regulated while interstitial marker (N-cadherin) expression was significantly up-regulated after LTF overexpression (Figs. 3A–3C). LTF knockdown can reverse EMT changes in tumor cells.

### LTF inhibits ferroptosis by suppressing the accumulation of Fe^2+^ within NSCLC cells

**Figure 4:**
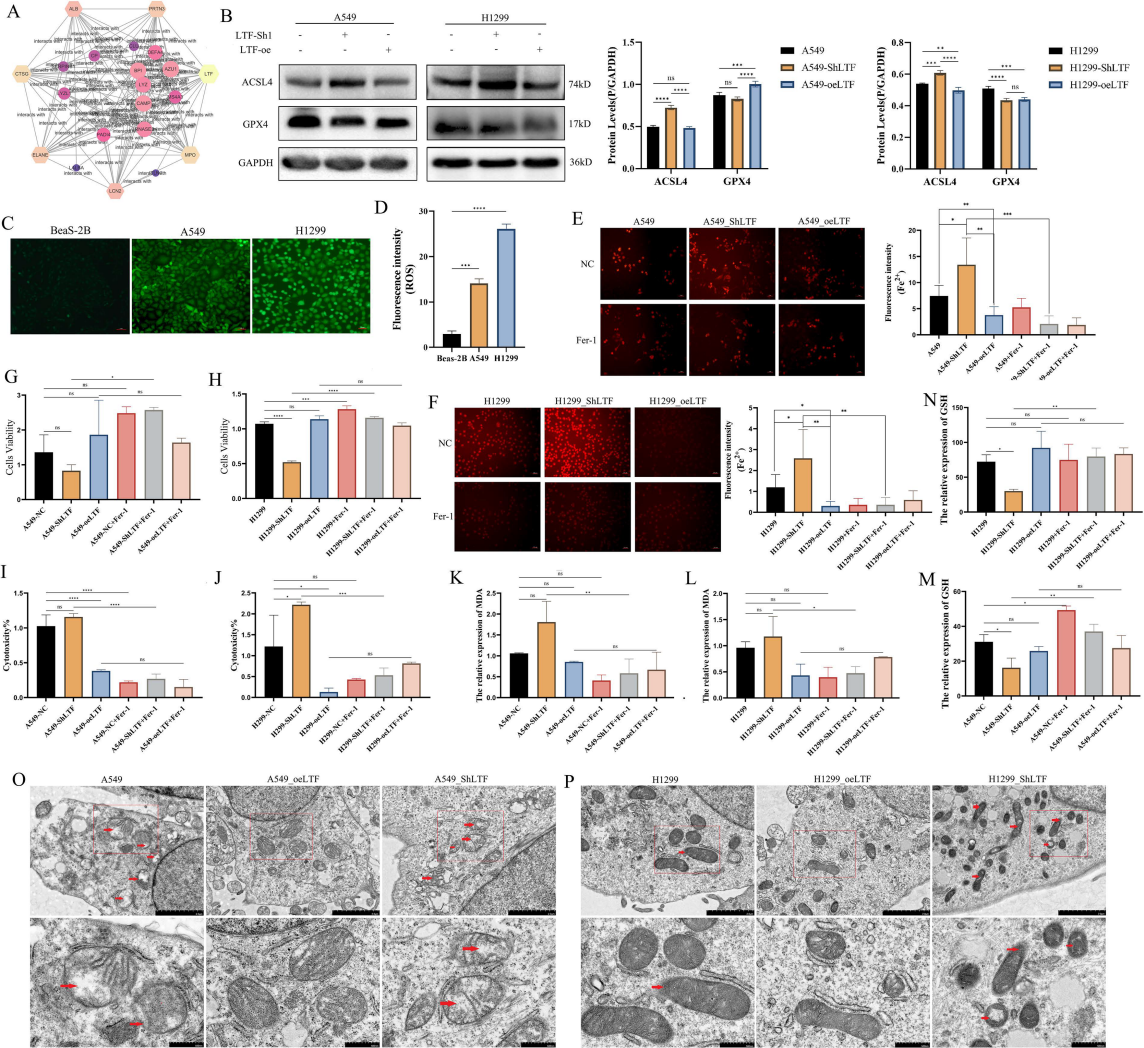
LTF is a key regulator of ferroptosis in NSCLC. (A) The PPI network of LTF to analyze genes highly associated gene. (B) Western blotting showing different expression levels of ACSL4 and GPX4 in silenced and overex pressed LTF cells. (C) The fluorescent probe assay of ROS in silenced and overex pressed LTF A549 cells. (D) The fluorescent probe assay of ROS in silenced and overex pressed LTF H1299 cells. (E) The fluorescent probe assay of Fe^2+^ in silenced and overex pressed LTF A549 cells. (F) The fluorescent probe assay of Fe^2+^ in silenced and overex pressed LTF H1299 cells. (G, H) The CCK8 assay of silenced and overex pressed LTF cells to detect cell viability. (I, J) The LDH assay of silenced and overex pressed LTF cells to detect cytotoxicity. (K, L) The MDA level assay of silenced and overex pressed LTF cells. (M, N) The GSH level assay of silenced and overex pressed LTF cells. (O, P) Transmission electron microscopy showing the morphological characteristics of typical ferroptosis of silenced and overex pressed LTF A549 and H1299 cells. (*n* ≥ 3. The values are expressed as the means ± SD. *P < 0.05, **P < 0.01, ***P < 0.001 and ****P < 0.0001.)

Previous studies have suggested that LTF may play an important role as a chelator and transporter of Fe^2+^ ions in the occurrence of ferroptosis. In the current study, PPI network analysis was performed to analyze genes highly associated with LTF. A total of 25 interacting genes were identified. Among these genes, 10 were found to be hub genes with high degree centrality values. These hub genes are likely to be key regulators in the ferroptosis processes, including MPO, CP, and LCN2 (Fig. 4A). Previous studies have confirmed the critical role of LTF in ferroptosis (Wang et al., 2020), and the current study further explores whether LTF is a key molecule in the occurrence of ferroptosis specifically in NSCLC. First, the expression of ACSL4 and GPX4 was detected after stable LTF knockdown and overexpression, and the results showed that LTF knockdown significantly promoted the expression of ACSL4 and inhibited the expression of GPX4, while the opposite pattern was demonstrated in the LTF overexpression group (Fig. 4B). Next, it was observed that intracellular Fe^2+^ levels were significantly decreased after LTF overexpression and increased significantly after LTF knockdown (Figs. 4E, 4F). The ROS levels of A549 cells and H1299 cells were significantly higher than those of Beas-2B cells(Figs. 4C, 4D). Additionally, the MDA levels were significantly elevated by LTF downregulation in A549 and H1299 cells and significantly decreased by LTF upregulation (Figs. 4K, 4L). GSH levels were significantly decreased by LTF downregulation in A549 and H1299 cells and significantly elevated by LTF upregulation (Figs. 4N, 4M). Lastly, the CCK-8 assay and LDH assay showed changes in cell activity and cytotoxicity after various treatments. As shown in Figs. 4G–4J, stable overexpression of LTF markedly accelerated cell activity and decelerated cell cytotoxicity. On the contrary, stable downregulation of LTF markedly decelerated cell activity and accelerated cell cytotoxicity, but this effect was blocked by ferrostatin-1. The morphological characteristics of typical ferroptosis, including mitochondrial atrophy, shrinkage, and increased membrane density, were observed using transmission electron microscopy in LTF knockdown and LTF overexpressed A549 and H1299 cells (Figs. 4O, 4P). These results indicated that LTF downregulation promoted ferroptosis in A549 and H1299 cells.

### lTF was overexpressed in peripheral blood of patients with NSCLC

**Figure 5:**
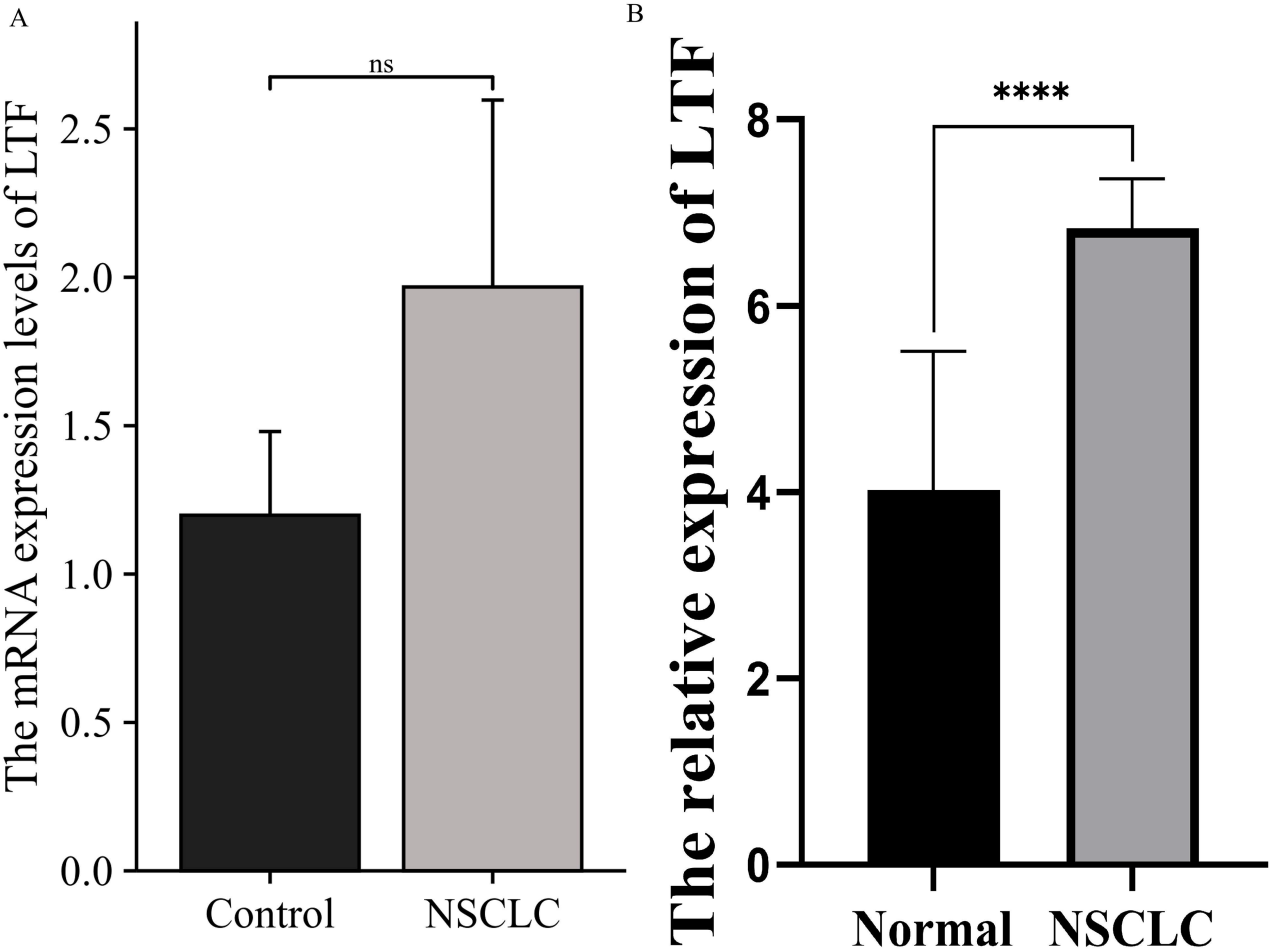
LTF was overexpressed in peripheral blood of patients with NSCLC. (A) The mRNA expression level of LTF in peripheral blood of normal and NSCLC. (B) The expression level of LTF protein in peripheral blood of normal and NSCLC. (*n* ≥ 3. The values are expressed as the means ± SD. ****P < 0.0001.)

To explore the influence of LTF as a key factor in the clinical features of NSCLC, PCR and ELISA were performed on clinically collected peripheral blood of normal and NSCLC patients to detect the expression of LTF mRNA and protein levels. The results showed that, compared with the normal group, the expression level of LTF protein was significantly increased in NSCLC group, while the change in mRNA level was not statistically significant (Fig. 5).

Patients with NSCLC were stratified into two groups based on LTF expression levels, allowing for an analysis of the correlation between LTF expression and various clinicopathological characteristics. The findings indicated a significant association between elevated LTF expression and disease stage (*P* = 0.0003), T stage (*P* = 0.0065), and N stage (*P* = 0.0099; Table 1).

**Table 1:** The association between the expression of LTF and clinical pathological features.

Characteristics		LTF		X^2^/U	*P*
		Low (19)	High (17)		
Gender	Female	11	5	2.948	0.086
	Male	8	12		
Age	<60	5	5	0.0429	0.836
	≥60	14	12		
Smoking	Yes	8	8	0.0892	0.765
	No	11	9		
Disease stage	I	15	5	58.50	0.0003*
	II	3	4		
	III	0	8		
	IV	0	0		
T stage	1	9	3	76.50	0.0065*
	2	8	8		
	3		5		
	4		1		
N stage	0	15	6	86.5	0.0099*
	1	3	6		
	2	1	5		
M stage	0	19	14	Ns	Ns
	1	0	3		

**Notes:**

Statistical analyses were performed using the Chi-square test in categorical variables (Gender, Age, and Smoking) and the Mann-Whitney U test in ordered categorical variable (Disease stage, T stage and N stage).

* *P* < 0.05 was considered significant.

## Discussion

The current landscape of non-small cell lung cancer (NSCLC) research highlights the critical role of various molecular pathways in tumor progression and treatment resistance (Riely et al., 2024). Existing methods primarily focus on targeting conventional pathways, yet the intricate interplay between iron metabolism and cancer cell survival remains underexplored. Recent findings suggest that ferroptosis, a form of regulated cell death driven by iron-dependent lipid peroxidation, is significantly implicated in the pathophysiology of NSCLC (Liao et al., 2024; Pan et al., 2024; Luo & Xu, 2022). Recent research has highlighted LTF, an important iron-binding glycoprotein, as a potential player in cancer biology, particularly in modulating cellular processes such as proliferation, apoptosis, and metastasis (Zhang et al., 2022; Zhao, Cheng & Xiong, 2021; Qi et al., 2021). Therefore, understanding the underlying mechanisms of NSCLC advancement is critical, particularly how LTF may influence tumor biology through ferroptosis, which has emerged as an important mechanism linked to cancer invasion and metastasis.

LTF is a multifunctional glycoprotein belonging to the transferrin family, primarily found in milk and other secretory fluids. It plays a crucial role in various biological processes including iron homeostasis, immune response, and inflammation regulation (Lu et al., 2025; Madkhali et al., 2023; Li et al., 2024a). LTF exhibits antimicrobial properties, contributing to host defense against pathogens by sequestering iron, which is essential for microbial growth. Additionally, LTF has been implicated in modulating cell proliferation and apoptosis, suggesting its potential role in cancer biology (Zheng et al., 2021). By using a combination of cell culture, molecular biology techniques, and bioinformatics analyses, this research provides a comprehensive assessment of LTF’s influence on iron metabolism and related signaling pathways in NSCLC. First, it was observed that LTF was upregulated in NSCLC. The findings indicate that LTF overexpression not only promotes the migration and invasion of NSCLC cells but also influences epithelial-mesenchymal transition (EMT), a critical process in cancer metastasis. Previous studies have confirmed the correlation between LTF and tumor migration, invasion, and EMT (Chiu et al., 2020). Moreover, the inhibition of LTF through knockdown experiments demonstrated a marked reduction in both the migration and invasion capabilities of NSCLC cells, highlighting the potential of LTF as a molecular target. This suggests that LTF may modulate the expression of key molecules involved in these pathways, thereby enhancing the aggressive characteristics of NSCLC.

The modulation of LTF levels can influence the availability of free iron, thereby impacting the ferroptosis process (Wang et al., 2020, 2024). LTF can sequester iron, reducing its availability for the Fenton reaction, which generates reactive oxygen species (ROS) that drive ferroptosis (Leveugle et al., 1994). The relationship between LTF and ferroptosis is particularly noteworthy, as our data demonstrate that LTF upregulation significantly inhibits ferroptosis, as evidenced by the fact that LTF can reduce the accumulation of intracellular iron ions (Fe^2+^). Therefore, this study examined the iron metabolism and oxidative stress levels of tumor cells after LTF silencing and overexpression, and the results showed that silencing LTF significantly enhanced these responses, while overexpression of LTF could reverse these responses. By controlling iron levels, LTF indirectly affects the expression of key ferroptosis-related genes, such as those involved in lipid metabolism and antioxidant defense mechanisms (Zeng et al., 2024). The current study indicates that LTF is associated with the expression of ferroptosis-related pathways, notably GPX4 and ACSL4. This indicates that LTF may exert its effects on NSCLC progression by modulating ferroptosis-related pathways, thus providing insights into potential therapeutic strategies targeting LTF to mitigate NSCLC advancement.

In addition, this study also examined the expression levels of LTF mRNA and protein in the peripheral blood of healthy controls and patients with NSCLC, as well as the correlation between LTF expression differences with TNM stages. The results showed that LTF protein levels, but not mRNA levels, were significantly different between NSCLC and healthy controls, and differences in protein levels were significantly correlated with the TNM stage of patients. Previous studies have primarily focused on gene expression levels, but the current findings suggest that protein expression may be a more relevant marker for assessing disease progression and prognosis in NSCLC. In addition to gene transcription, protein post-translational modifications may also affect protein expression including at the functional level, such as acetylation (He, Li & Li, 2023; Zhuo et al., 2023) and SUMOylation (Wang et al., 2024). The stability of LTF protein could be modulated by ubiquitin-proteasome pathways in tumor microenvironments (Wang et al., 2024, 2020). Further studies are needed to dissect the dominant mechanism of this process. This is consistent with the work of Zhang et al. (2013) which indicated that protein isoform profiles can provide critical insights that gene expression levels alone may overlook. Furthermore, the current study is among the first to establish a direct link between LTF protein levels and the TNM staging system, suggesting that LTF shows potential as a candidate biomarker for NSCLC, but further validation in larger cohorts is warranted.

The present study acknowledges certain limitations that warrant consideration. The sample size may not be sufficiently large enough to generalize findings across diverse populations, potentially affecting the external validity of results. Besides, the lack of significance at the mRNA level despite protein-level changes suggests post-transcriptional regulation or technical variability, requiring further investigation. Additionally, the mechanisms behind LTF modulation of ferroptosis in non-small cell lung cancer (NSCLC) remain inadequately explored. Our experimental conclusion suggests that LTF inhibits ferroptosis by regulating the accumulation of Fe^2+^ within cells, thereby indirectly affecting the expression of GPX4 and ACSL4. However, the specific molecular mechanism of the correlation between LTF and GPX4 as well as ACSL4 indeed requires further verification. In the future, this conclusion can be refined through studies such as ubiquitination experiments, chromatin immunoprecipitation (ChIP), or co-immunoprecipitation (co-IP).

LTF upregulation significantly influences the pathological changes in non-small cell lung cancer through the modulation of ferroptosis. This research elucidates the molecular mechanisms involved in LTF’s regulatory effects, highlighting its potential role as a therapeutic target in combating cancer progression. The findings underscore the importance of ferroptosis as a key player in the disease’s pathology, suggesting that enhancing LTF expression may offer a novel strategy for intervention in non-small cell lung cancer. Overall, the study contributes valuable insights into the interplay between LTF and ferroptosis in oncological contexts.

## Conclusions

In conclusion, this study has demonstrated that the upregulation of LTF plays a crucial role in inducing the EMT transformation of NSCLC cells by inhibiting the ferroptosis process of tumor cells, thereby promoting their migration and invasion. LTF is linked to key ferroptosis regulators such as GPX4 and ACSL4, ultimately leading to altered redox status in cancer cells. Notably, elevated LTF levels in peripheral blood correlate with NSCLC stages, highlighting LTF’s potential as a candidate biomarker for NSCLC. This research underscores the significance of targeting ferroptosis as a promising therapeutic strategy in NSCLC management, paving the way for future investigations into LTF’s mechanistic roles.

## Supplemental Information

10.7717/peerj.20866/supp-1 Supplemental Information 1 Original data of Western blotting.

10.7717/peerj.20866/supp-2 Supplemental Information 2 Original data of ELISA 1.

10.7717/peerj.20866/supp-3 Supplemental Information 3 Original data of ELISA 2.

10.7717/peerj.20866/supp-4 Supplemental Information 4 Original data of clinical trials.

10.7717/peerj.20866/supp-5 Supplemental Information 5 Details of PCR data analysis.

10.7717/peerj.20866/supp-6 Supplemental Information 6 Specific details of the qPCR experiment.

10.7717/peerj.20866/supp-7 Supplemental Information 7 MIQE checklist.

10.7717/peerj.20866/supp-8 Supplemental Information 8 Detection of cell phenotypes after transfection of cells with negative control lentivirus. A, B: The CCK8 assay was performed on negative control lentivirus-transfected cells to detect cell viability. C, D: The LDH assay was performed on negative control lentivirus-transfected cells to detect cytotoxicity. E, F: The Fluorescent probe assay of Fe 2+ in negative control lentivirus-transfected cells . G, H: The Fluorescent probe assay of ROS in negative control lentivirus-transfected cells . I: Transwell experiment showing different infiltration of negative control lentivirus-transfected cells.

10.7717/peerj.20866/supp-9 Supplemental Information 9 Cell Transfection Lentivirus Outbound Form of Keji Biotechnology Co., Ltd.

10.7717/peerj.20866/supp-10 Supplemental Information 10 Translation Codebook for Cell Transfection Lentivirus Outbound Form.

10.7717/peerj.20866/supp-11 Supplemental Information 11 Positive controls of ROS, Fe2+ and TEM imaging. 1.ROS Probe (DCFH-DA/C11-BODIPY) Assay: Positive Control: Beas-2B Cells treated with100 μM tert-butyl hydroperoxide (TBHP)for 2 h (known ROS inducer) were used alongside experimental groups (Supplementary Figure S3A). This treatment induced significantly increasein DCF fluorescence *vs*. untreated cells (p<0.001), validating probe sensitivity. 2. Fe²⁺ Probe (FerroOrange/RPA) Staining: Positive Control: Cells incubated with10 μM Erastinfor 24 showed asignificant FerroOrange signal boost(Supplementary Figure S3B). This confirmed Fe²⁺-specific detection under our experimental conditions. 3. TEM Imaging: Positive Control: Cells treated with10 μM Erastinfor 24 h (classic ferroptosis inducer) exhibitedcharacteristic mitochondrial shrinkage and increased membrane density, distinct from apoptosis/necrosis (Supplementary Figure S3C). These features matched prior report.

10.7717/peerj.20866/supp-12 Supplemental Information 12 Supplementary Materials Template for Lentiviral Vector Details. A,B: The MOI of A549 and H1299 cells were determined by pilot transduction with GFP reporter virus. C,D: The lentiviral transfection efficiencies of A549 cells and H1299 cells

10.7717/peerj.20866/supp-13 Supplemental Information 13 Multivariate Logistic Regression Analysis of LTF Association With Advanced TNM Stage Adjusted for Clinical Confounders. Multivariate Logistic Regression Analysis of LTF Association With Advanced TNM Stage Adjusted for Clinical Confounders

10.7717/peerj.20866/supp-14 Supplemental Information 14 ELISA and PCR results were obtained for each corresponding sample in the split sample.

10.7717/peerj.20866/supp-15 Supplemental Information 15 Supplementary Materials Template for Lentiviral Vector Details and Positive controls of ROS, Fe2+ and TEM imaging.

## Abbreviations

NSCLC: Non-small Cell Lung Cancer
TCGA: The Cancer Genome Atlas
LTF: Lactotransferrin
EMT: Epithelial-mesenchymal Transition

## Additional Information and Declarations

### Data Availability

The following information was supplied regarding data availability:

The raw data is available in the Supplemental Files.

## References

[ref-1] Chiu IJ, Hsu YH, Chang JS, Yang JC, Chiu HW, Lin YF (2020). Lactotransferrin downregulation drives the metastatic progression in clear cell renal cell carcinoma. Cancers.

[ref-2] Golabek K, Raczka G, Gazdzicka J, Miskiewicz-Orczyk K, Zieba N, Krakowczyk L, Hudy D, Asman M, Misiolek M, Strzelczyk JK (2022). Single nucleotide polymorphism and mRNA expression of LTF in oral squamous cell carcinoma. Genes.

[ref-3] Habib CN, Ali AE, Anber NH, George MY (2023). Lactoferrin ameliorates carfilzomib-induced renal and pulmonary deficits: insights to the inflammasome NLRP3/NF-kappaB and PI3K/Akt/GSK-3beta/MAPK axes. Life Sciences.

[ref-4] He W, Li Q, Li X (2023). Acetyl-CoA regulates lipid metabolism and histone acetylation modification in cancer. Biochimica et Biophysica Acta-Reviews on Cancer.

[ref-5] Iijima H, Tomizawa Y, Iwasaki Y, Sato K, Sunaga N, Dobashi K, Saito R, Nakajima T, Minna JD, Mori M (2006). Genetic and epigenetic inactivation of LTF gene at 3p21.3 in lung cancers. International Journal of Cancer.

[ref-6] Leveugle B, Spik G, Perl DP, Bouras C, Fillit HM, Hof PR (1994). The iron-binding protein lactotransferrin is present in pathologic lesions in a variety of neurodegenerative disorders: a comparative immunohistochemical analysis. Brain Research.

[ref-7] Li S, He Y, Chen K, Sun J, Zhang L, He Y, Yu H, Li Q (2021). RSL3 drives ferroptosis through NF-kappaB pathway activation and GPX4 depletion in glioblastoma. Oxidative Medicine and Cellular Longevity.

[ref-8] Li NC, Iannuzo N, Christenson SA, Langlais PR, Kraft M, Ledford JG, Li X (2024a). Investigation of lactotransferrin messenger RNA expression levels as an anti-type 2 asthma biomarker. Journal of Allergy and Clinical Immunology.

[ref-9] Li C, Lei S, Ding L, Xu Y, Wu X, Wang H, Zhang Z, Gao T, Zhang Y, Li L (2023). Global burden and trends of lung cancer incidence and mortality. Chinese Medical Journal.

[ref-10] Li Q, Song Q, Pei H, Chen Y (2024b). Emerging mechanisms of ferroptosis and its implications in lung cancer. Chinese Medical Journal.

[ref-11] Liao W, Zhang R, Chen G, Zhu X, Wu W, Chen Z, Jiang C, Lin Z, Ma L, Yu H (2024). Berberine synergises with ferroptosis inducer sensitizing NSCLC to ferroptosis in p53-dependent SLC7A11-GPX4 pathway. Biomedicine & Pharmacotherapy.

[ref-12] Liu W, Sun Y, Huo Y, Zhang L, Zhang N, Yang M (2024). Circular RNAs in lung cancer: implications for preventing therapeutic resistance. EBioMedicine.

[ref-13] Liu X, Wang Z, Liu M, Zhi F, Wang P, Liu X, Yu S, Liu B, Jiang Y (2022). Identification of LTF as a prognostic biomarker for osteosarcoma. Journal of Oncology.

[ref-14] Lu J, Liu S, Wei M, Zhang W, Zhu T, Xing L, Liu J, Zheng X, Pang X, Zhang S, Lv J (2025). The impact of heating-induced lactosylation on the digestibility of lactotransferrin. Food Chemistry.

[ref-15] Luo L, Xu G (2022). Fascaplysin induces apoptosis and ferroptosis, and enhances Anti-PD-1 immunotherapy in non-small cell lung cancer (NSCLC) by promoting PD-L1 expression. International Journal of Molecular Sciences.

[ref-16] Madkhali OA, Moni SS, Sultan MH, Bakkari MA, Almoshari Y, Shaheen ES, Alshammari A (2023). Design and characterization of Lactotransferrin peptide-loaded dextran-docosahexaenoic acid nanoparticles: an immune modulator for hepatic damage. Scientific Reports.

[ref-17] Pan Z, Li B, Lu P, Rong G, Wang X (2024). Inhibiting LCN2 can suppress the development of NSCLC by promoting ferroptosis. Gene.

[ref-18] Qi YF, Yang Y, Zhang Y, Liu S, Luo B, Liu W (2021). Down regulation of lactotransferrin enhanced radio-sensitivity of nasopharyngeal carcinoma. Computational Biology and Chemistry.

[ref-100] R Core Team (2022). R: a language and environment for statistical computing.

[ref-19] Riely GJ, Wood DE, Ettinger DS, Aisner DL, Akerley W, Bauman JR, Bharat A, Bruno DS, Chang JY, Chirieac LR, DeCamp M, Desai AP, Dilling TJ, Dowell J, Durm GA, Gettinger S, Grotz TE, Gubens MA, Juloori A, Lackner RP, Lanuti M, Lin J, Loo BW, Lovly CM, Maldonado F, Massarelli E, Morgensztern D, Mullikin TC, Ng T, Owen D, Owen DH, Patel SP, Patil T, Polanco PM, Riess J, Shapiro TA, Singh AP, Stevenson J, Tam A, Tanvetyanon T, Yanagawa J, Yang SC, Yau E, Gregory KM, Hang L (2024). Non-small cell lung cancer, version 4.2024, NCCN clinical practice guidelines in oncology. Journal of the National Comprehensive Cancer Network.

[ref-20] Wang Y, Liu Y, Liu J, Kang R, Tang D (2020). NEDD4L-mediated LTF protein degradation limits ferroptosis. Biochemical and Biophysical Research Communications.

[ref-21] Wang J, Xiu M, Wang J, Gao Y, Li Y (2024). METTL16-SENP3-LTF axis confers ferroptosis resistance and facilitates tumorigenesis in hepatocellular carcinoma. Journal of Hematology & Oncology.

[ref-22] Wen J, Zheng W, Zeng L, Wang B, Chen D, Chen Y, Lu X, Shao C, Chen J, Fan M (2023). LTF induces radioresistance by promoting autophagy and forms an AMPK/SP2/NEAT1/miR-214-5p feedback loop in lung squamous cell carcinoma. International Journal of Biological Sciences.

[ref-23] Xiao H, Wang K, Li D, Wang K, Yu M (2021). Evaluation of FGFR1 as a diagnostic biomarker for ovarian cancer using TCGA and GEO datasets. PeerJ.

[ref-24] Yami HA, Tahmoorespur M, Javadmanesh A, Tazarghi A, Sekhavati MH (2023). The immunomodulatory effects of lactoferrin and its derived peptides on NF-kappaB signaling pathway: a systematic review and meta-analysis. Immunity Inflammation and Disease.

[ref-25] Yao F, Deng Y, Zhao Y, Mei Y, Zhang Y, Liu X, Martinez C, Su X, Rosato RR, Teng H, Hang Q, Yap S, Chen D, Wang Y, Chen MM, Zhang M, Liang H, Xie D, Chen X, Zhu H, Chang JC, You MJ, Sun Y, Gan B, Ma L (2021). A targetable LIFR-NF-kappaB-LCN2 axis controls liver tumorigenesis and vulnerability to ferroptosis. Nature Communications.

[ref-26] Zeng C, Gu X, Chen Y, Lin Y, Chen J, Chen Z, Chen C, Yao G, Lin C (2024). Identification and experimental validation of ferroptosis-related gene lactotransferrin in age-related hearing loss. Frontiers in Aging Neuroscience.

[ref-27] Zhang J, Liu S, Chen X, Xu X, Xu F (2023). Non-immune cell components in tumor microenvironment influencing lung cancer Immunotherapy. Biomedicine & Pharmacotherapy.

[ref-28] Zhang Z, Pal S, Bi Y, Tchou J, Davuluri RV (2013). Isoform level expression profiles provide better cancer signatures than gene level expression profiles. Genome Medicine.

[ref-29] Zhang XB, Xu SQ, Hui YG, Zhou HY, Hu YC, Zhang RH, Gao XD, Zheng CM (2022). Lactotransferrin promotes intervertebral disc degeneration by regulating Fas and inhibiting human nucleus pulposus cell apoptosis. Aging.

[ref-30] Zhao Q, Cheng Y, Xiong Y (2021). LTF regulates the immune microenvironment of prostate cancer through JAK/STAT3 pathway. Frontiers in Oncology.

[ref-31] Zheng JQ, Lin CH, Lee HH, Wang WK, Tong YS, Lee KY, Chiu HW, Lin YF (2021). Lactotransferrin downregulation serves as a potential predictor for the therapeutic effectiveness of mTOR inhibitors in the metastatic clear cell renal cell carcinoma without PTEN mutation. Biomedicines.

[ref-32] Zhuo FF, Li L, Liu TT, Liang XM, Yang Z, Zheng YZ, Luo QW, Lu JH, Liu D, Zeng KW, Tu PF (2023). Lycorine promotes IDH1 acetylation to induce mitochondrial dynamics imbalance in colorectal cancer cells. Cancer Letters.

