# Supplementary material for: Lactotransferrin upregulation affects the pathological changes of non-small cell lung cancer by regulating ferroptosis
Source: PeerJ. 2026 Feb 27;14:e20866. doi: 10.7717/peerj.20866 (PMC12951881; doi:10.7717/peerj.20866)
Supplement: Supplemental Information 6 [file peerj-14-20866-s006.docx]

**RNA quantification and purity detection results**

| Sample Number | RNA concentration (ng/ul) | A260/A280 | A260/A230 |
| --- | --- | --- | --- |
| Normal1 | 136.73 | 1.98 | 2.05 |
| Normal2 | 143.89 | 1.86 | 2.03 |
| Normal3 | 134.98 | 1.95 | 2.01 |
| Normal4 | 112.10 | 1.96 | 2.04 |
| Normal5 | 116.98 | 1.89 | 1.98 |
| Normal6 | 125.89 | 1.89 | 1.89 |
| NSCLC1 | 109.69 | 1.87 | 1.97 |
| NSCLC2 | 124.59 | 1.97 | 2.06 |
| NSCLC3 | 134.98 | 1.94 | 1.97 |
| NSCLC4 | 143.90 | 1.93 | 2.01 |
| NSCLC5 | 111.53 | 1.96 | 2.03 |
| NSCLC6 | 116.78 | 1.98 | 2.04 |

**Reverse transcription (PrimeScriptTM RT kit, Takara Bio)**

According to the instructions, set the reaction system and reaction conditions, and reverse transcribe RNA into cDNA.

1) Removal of genomic DNA: Prepare the following mixture in an enzyme-free EP tube, pipette and mix well, at 42 ° C for 2 minutes.

| Component | Volume |
| --- | --- |
| Total RNA * | 10 μL |
| 4 × gDNA wiper Mix | 4 μL |
| RNase-free ddH_2_O | Add to 16 μL |

2) Reverse transcription reaction: Add 5×HiScript III qRT SuperMix, blow and mix evenly, 37℃, 15 minutes; 85℃, 5 seconds.

3）Freeze the cDNA samples at -80℃ for later use

**qPCR reaction**

Take out the cDNA samples that have been frozen at -80℃. Place tubes on ice for immediate use. Vortex briefly for mix gently.

**1) Configure the reaction system as per the instructions, set the reaction conditions, and detect the current expression level of the gene**

**Primer sequences（5'-3')**

| Gene ID | Primer | Sequences (5'to3') |
| --- | --- | --- |
| GAPDH | F | GTGGACCTGACCTGCCGTCTAG |
|  | R | GAGTGGGTGTCGCTGTTGAAGTC |
| LTF | F | CCGCCGTGGACAGGACTG |
|  | R | CGCCAATACACAGAGCACAGAG |

**Prepare the reaction system**

| Component | Volume |
| --- | --- |
| 2 × AceQ Universal SYBR qPCR Master Mix | 10μl |
| Primer1(10 µM) | 0.4μl |
| Primer2(10 µM) | 0.4μl |
| Reverse transcription product（cDNA） | 2.0μl |
| ddH_2_O | To 20.0 µl |

**Experiment repetition: 3 Wells per sample.**

**Set reaction conditions**

| Stage | Steps | Number of cycles | Temperature | Time |
| --- | --- | --- | --- | --- |
| Stage1 | Pre-deformation | 1 | 95℃ | 5min |
| Stage2 | Cyclic reaction | 40 | 95℃ | 10s |
|  |  |  | 60℃ | 30s |
| Stage3 | Melting curve | 1 | 95℃ | 15s |
|  |  |  | 60℃ | 60s |
|  |  |  | 95℃ | 15s |

**Data analysis method**

The relative expression level of the target gene was analyzed using the 2-△△CT method.

1. Calculate the mean Ct value of each gene in each sample (the mean Ct value of each gene in each sample of each group).

2. Subtract the Ct value of the internal reference molecule in the same sample from the Ct value of the target molecule in each sample of each group to obtain the ∆Ct value of the target molecule in each sample of each group.

3. Calculate the arithmetic mean of the ∆Ct values of the target molecule in all samples of the control group.

4. Subtract the mean ∆Ct value of the target molecule in all samples of the control group (step 3) from the ∆Ct value of the target molecule in each sample of each group (step 2) to obtain the ∆∆Ct value of the target molecule in each sample of each group.

5. Calculate the relative expression level of the target molecule in each sample of each group by 2^-∆∆Ct based on the ∆∆Ct value of the target molecule in each sample of each group obtained in step 4.

The two-tailed Student’s t-test was performed to compare the differences between two groups.

**Visualization charts of RT-PCR experiments**


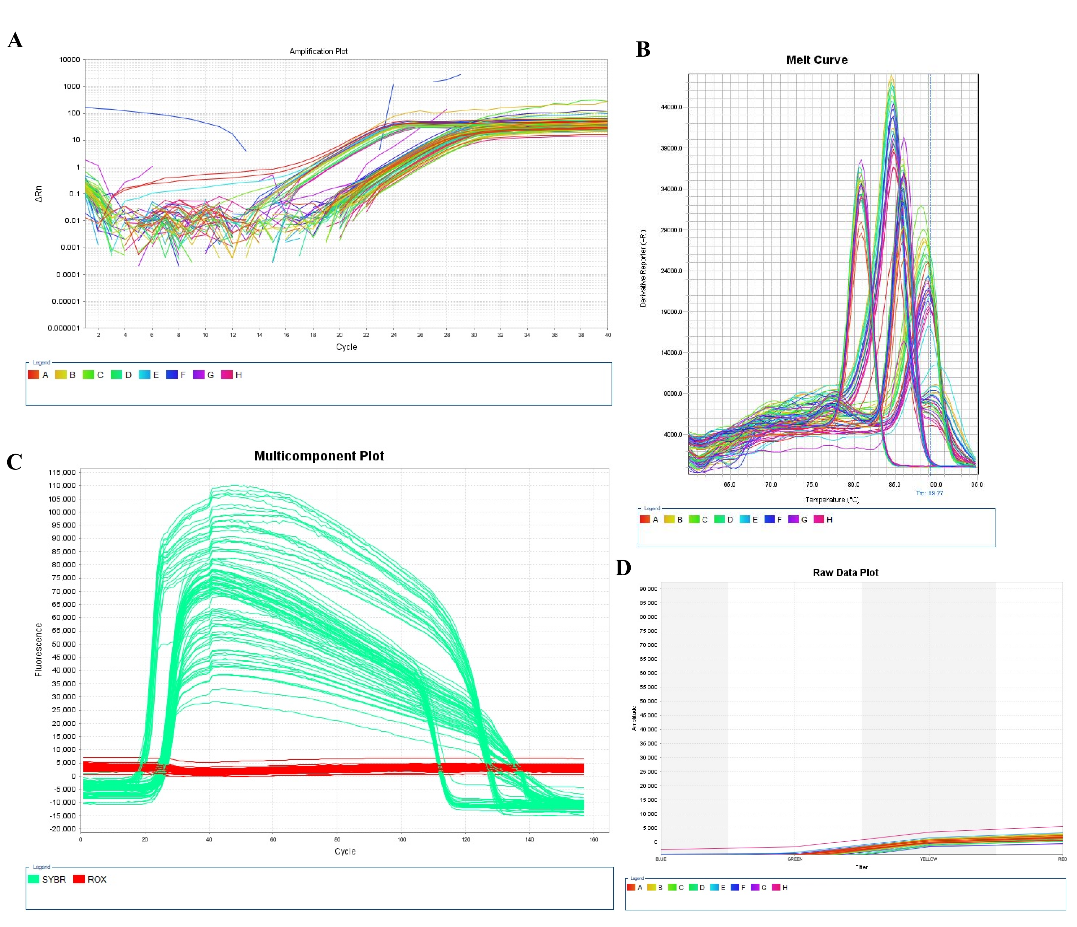
1. Visualization chart of PCR experiments for samples in the normal group

A: Amplification Plot; B: Melt Curve; C: Multi - component Plot; D:Raw Dataplot

2. Visualization chart of PCR experiments for samples in the NSCLC group


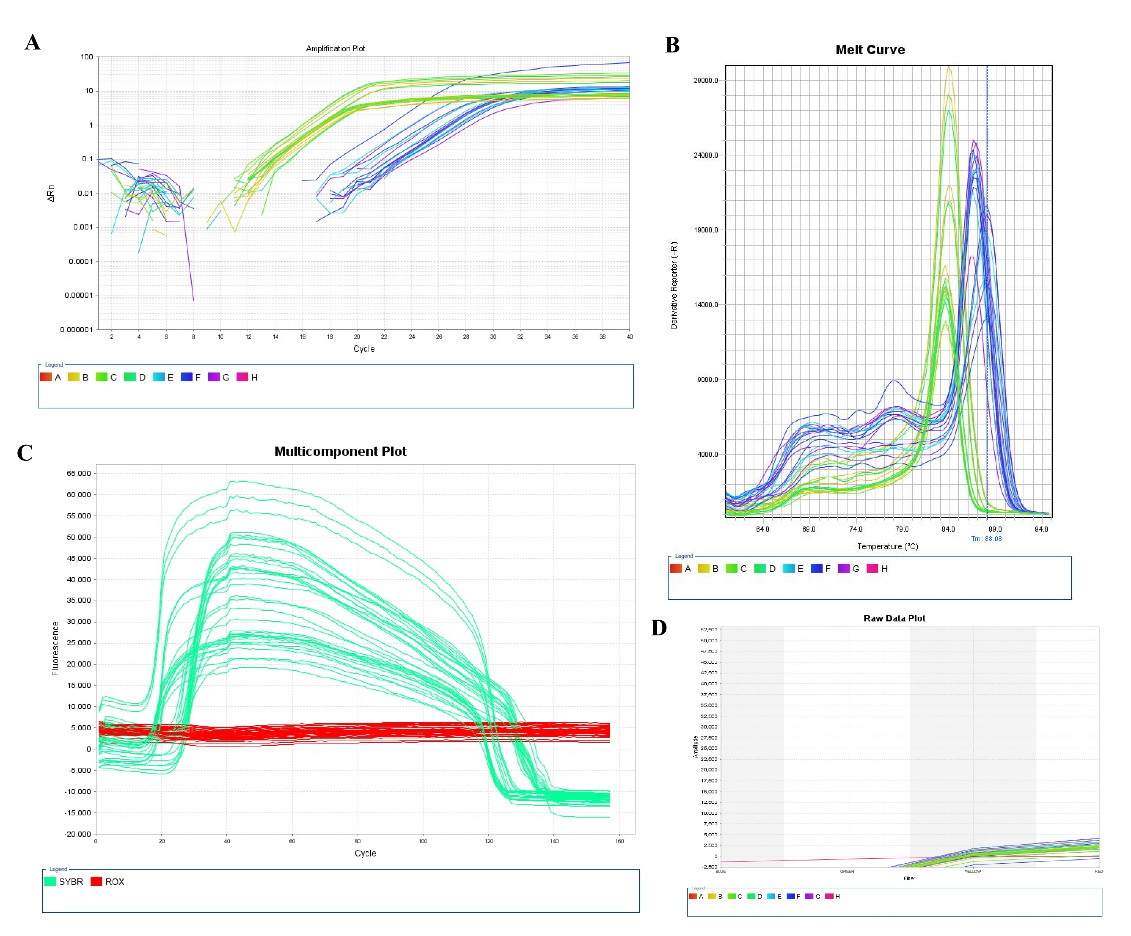
A: Amplification Plot; B: Melt Curve; C: Multi - component Plot; D:Raw Dataplot
