# Supplementary figures and images for: Lactotransferrin upregulation affects the pathological changes of non-small cell lung cancer by regulating ferroptosis
Source: PeerJ. 2026 Feb 27;14:e20866. doi: 10.7717/peerj.20866 (PMC12951881; doi:10.7717/peerj.20866)

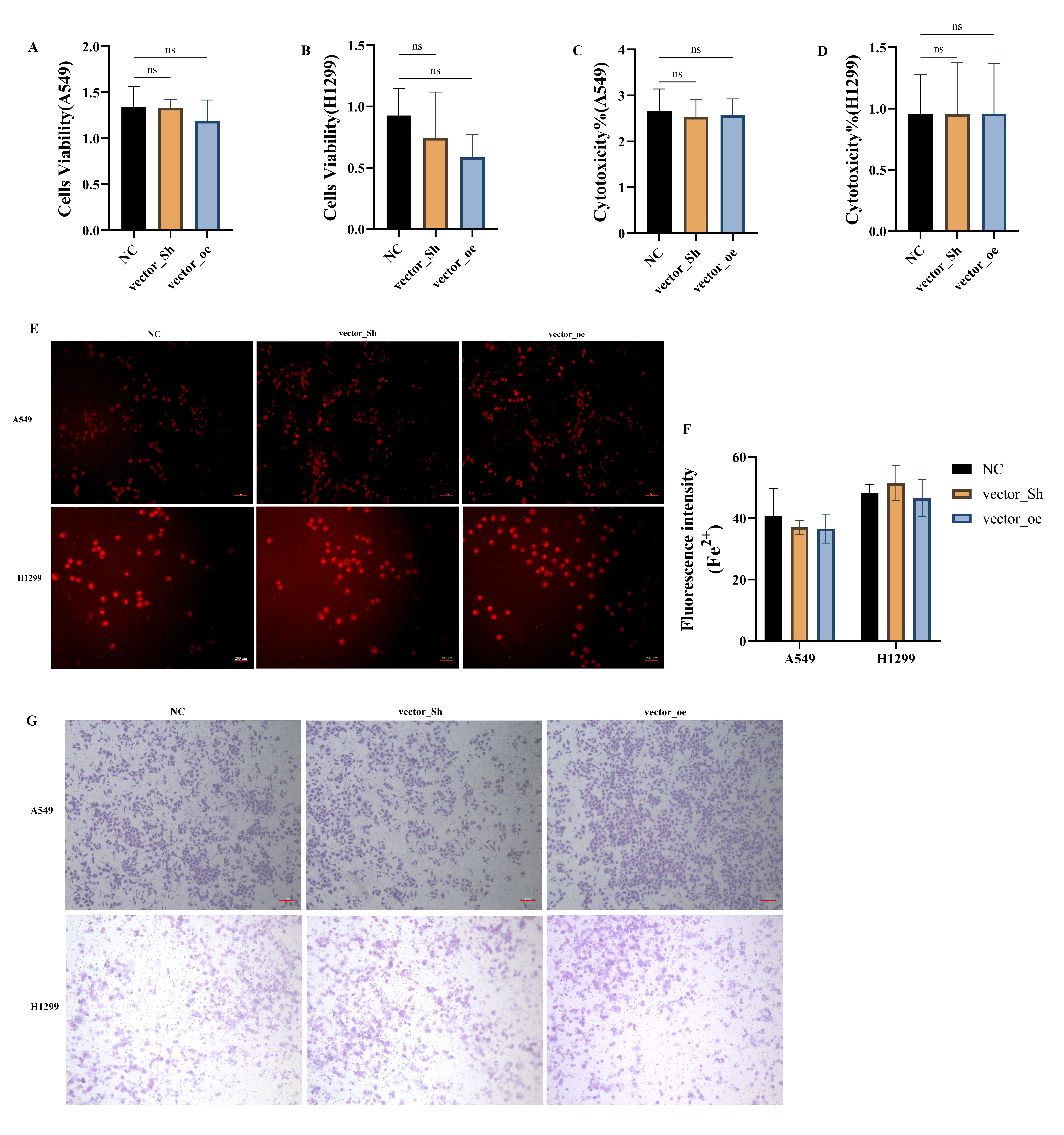

Supplement: Supplemental Information 8 — A, B: The CCK8 assay was performed on negative control lentivirus-transfected cells to detect cell viability. C, D: The LDH assay was performed on negative control lentivirus-transfected cells to detect cytotoxicity. E, F: The Fluorescent probe assay of Fe 2+ in negative control lentivirus-transfected cells . G, H: The Fluorescent probe assay of ROS in negative control lentivirus-transfected cells . I: Transwell experiment showing different infiltration of negative control lentivirus-transfected cells. [file peerj-14-20866-s008.tif]

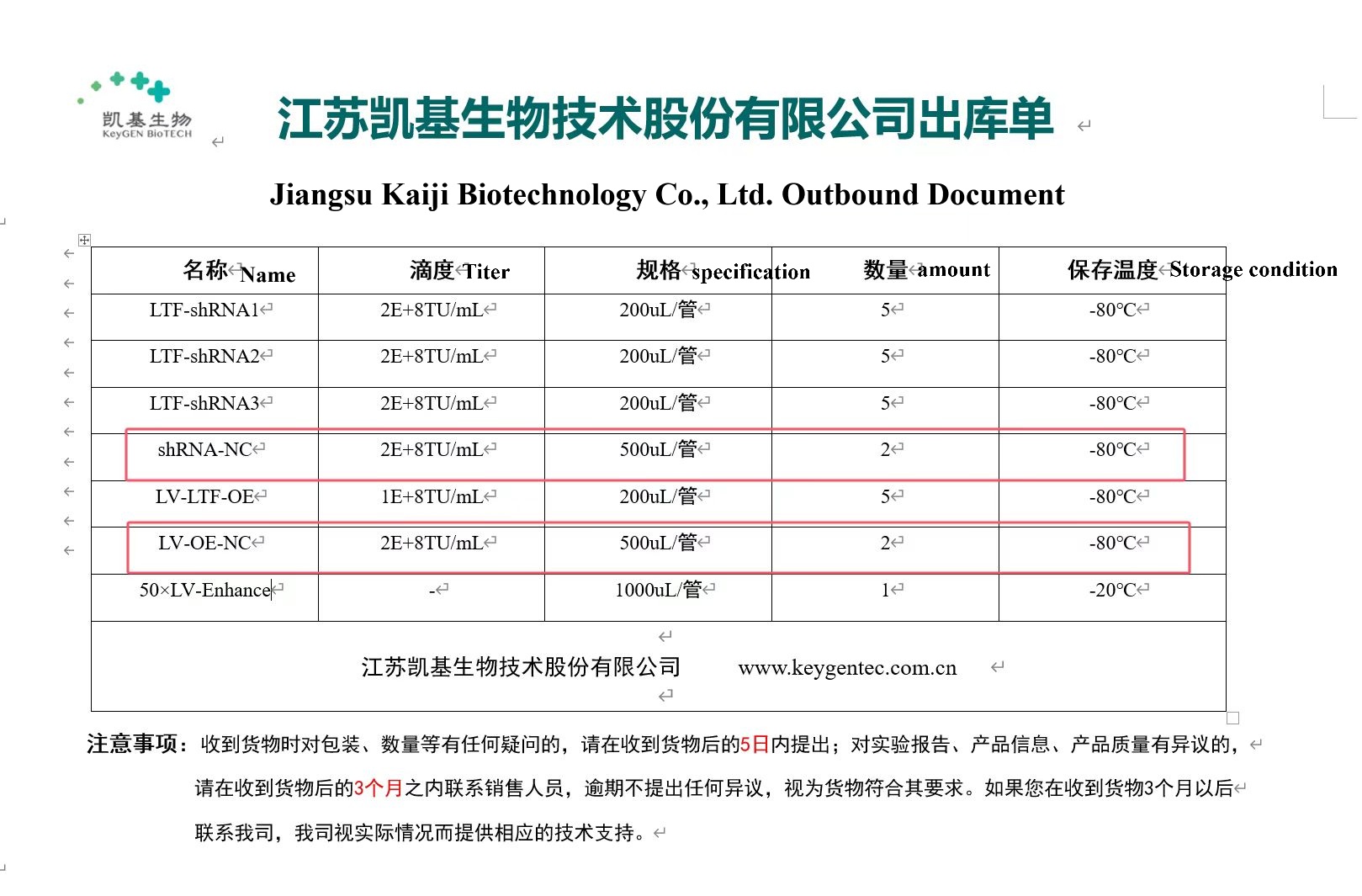

Supplement: Supplemental Information 9 [file peerj-14-20866-s009.jpg]

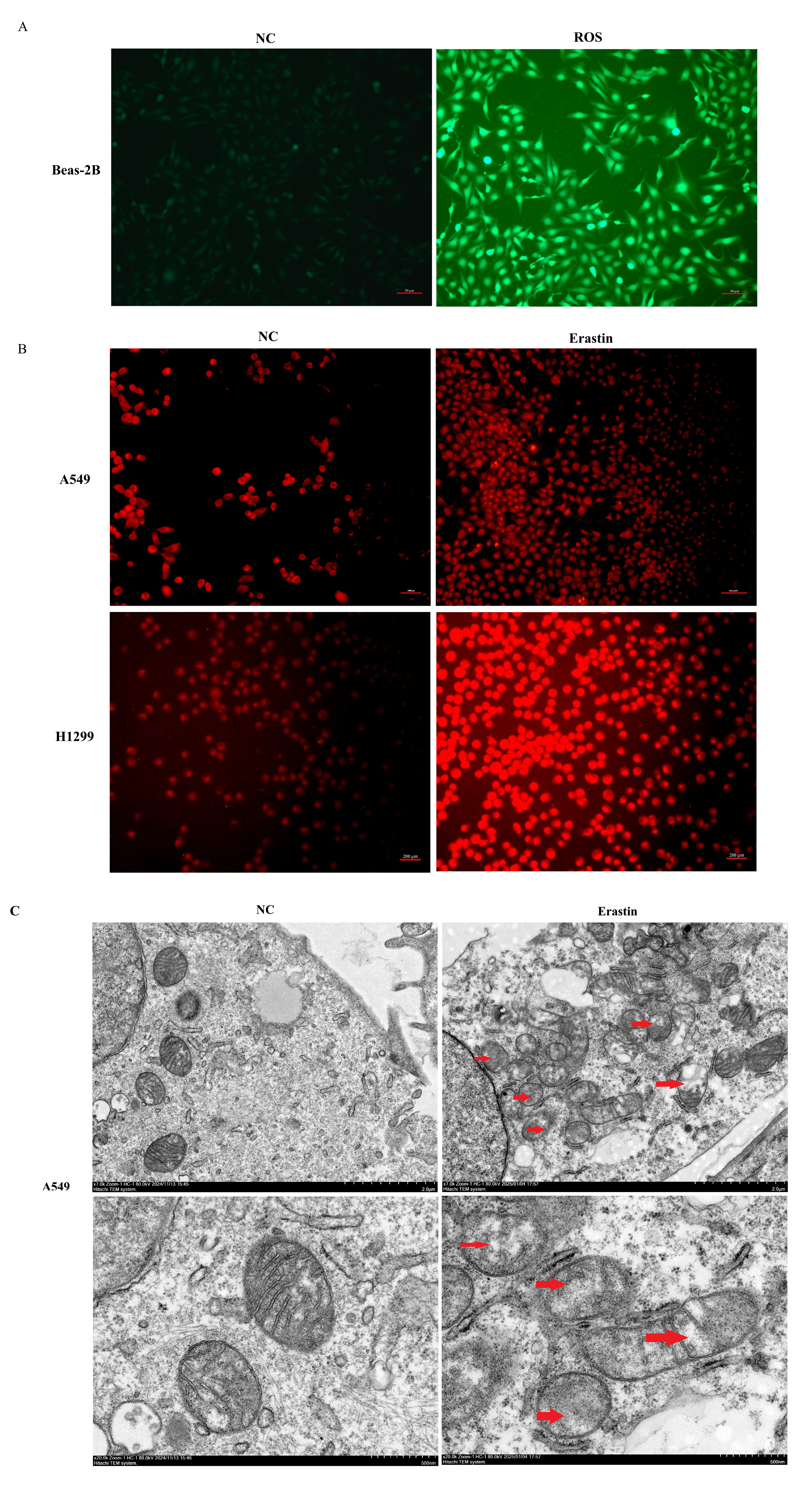

Supplement: Supplemental Information 11 — 1.ROS Probe (DCFH-DA/C11-BODIPY) Assay: Positive Control: Beas-2B Cells treated with100 μM tert-butyl hydroperoxide (TBHP)for 2 h (known ROS inducer) were used alongside experimental groups (Supplementary Figure S3A). This treatment induced significantly increasein DCF fluorescence vs. untreated cells (p<0.001), validating probe sensitivity. 2. Fe²⁺ Probe (FerroOrange/RPA) Staining: Positive Control: Cells incubated with10 μM Erastinfor 24 showed asignificant FerroOrange signal boost(Supplementary Figure S3B). This confirmed Fe²⁺-specific detection under our experimental conditions. 3. TEM Imaging: Positive Control: Cells treated with10 μM Erastinfor 24 h (classic ferroptosis inducer) exhibitedcharacteristic mitochondrial shrinkage and increased membrane density, distinct from apoptosis/necrosis (Supplementary Figure S3C). These features matched prior report. [file peerj-14-20866-s011.png]

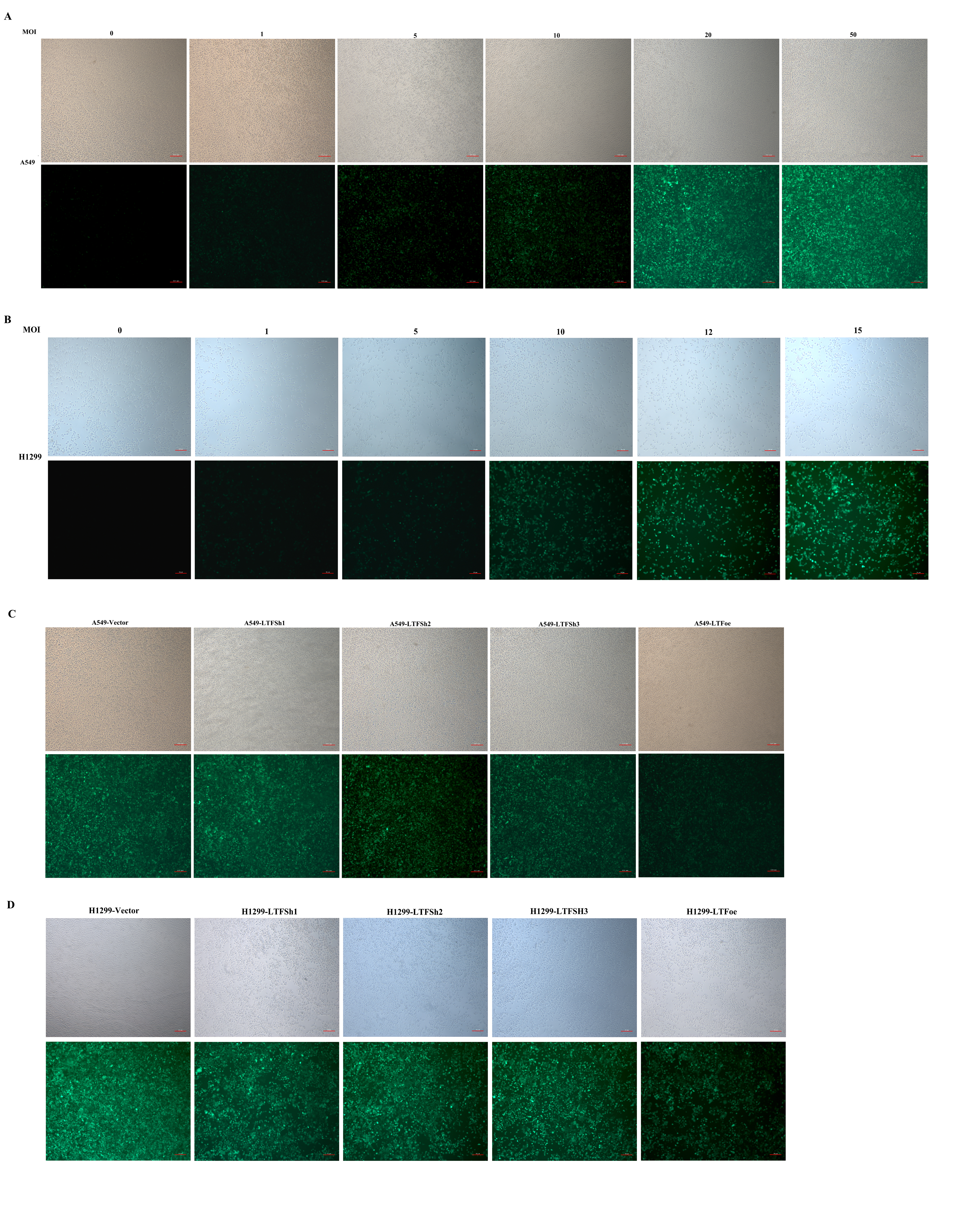

Supplement: Supplemental Information 12 — A,B: The MOI of A549 and H1299 cells were determined by pilot transduction with GFP reporter virus. C,D: The lentiviral transfection efficiencies of A549 cells and H1299 cells [file peerj-14-20866-s012.png]
