# Supplementary material for: Lactotransferrin upregulation affects the pathological changes of non-small cell lung cancer by regulating ferroptosis
Source: PeerJ. 2026 Feb 27;14:e20866. doi: 10.7717/peerj.20866 (PMC12951881; doi:10.7717/peerj.20866)
Supplement: Supplemental Information 10 [file peerj-14-20866-s010.docx]

**Jiangsu Kaiji Biotechnology Co,,Ltd. Outbound Document**

| Name | Titer | Specification | Amount | Storage condition |
| --- | --- | --- | --- | --- |
| LTF-shRNA1 | 2E+8TU/ml | 200ul/tube | 5 | -80℃ |
| LTF-shRNA2 | 2E+8TU/ml | 200ul/tube | 5 | -80℃ |
| LTF-shRNA3 | 2E+8TU/ml | 200ul/tube | 5 | -80℃ |
| shRNA-NC | 2E+8TU/ml | 500ul/tube | 2 | -80℃ |
| LV-LTF-OE | 1E+8TU/ml | 200ul/tube | 5 | -80℃ |
| LV-LTF-NC | 2E+8TU/ml | 500ul/tube | 2 | -80℃ |
| 50*LV-Enhance |  | 1000ul/tube | 1 | -20℃ |
| Jiangsu Kaiji Biotechnology Co,,Ltd. ww.kaygentec.com.cn | | | | |

**Important Notice:** For any issues related to packaging or quantity, please notify us within 5 days of receiving the goods.For disputes concerning test reports, product specifications, or product quality, please contact our sales team within 3 months of receipt. Failure to raise objections within this period will be considered as acceptance that the goods meet all requirements. If you contact us more than 3 months after delivery, we will evaluate the situation and may provide technical support on a case-by-case basis.
