# Supplementary material for: Lactotransferrin upregulation affects the pathological changes of non-small cell lung cancer by regulating ferroptosis
Source: PeerJ. 2026 Feb 27;14:e20866. doi: 10.7717/peerj.20866 (PMC12951881; doi:10.7717/peerj.20866)
Supplement: Supplemental Information 13 — Multivariate Logistic Regression Analysis of LTF Association With Advanced TNM Stage Adjusted for Clinical Confounders [file peerj-14-20866-s013.docx]

Multivariate logistic regression was performed to assess the independent association between LTF and advanced TNM stage (defined as III–IV vs. I–II), adjusting for potential confounders including ag, sex and smoking status. Results are reported as adjusted odds ratios (ORs) with 95% confidence intervals (CIs). Model fit was evaluated using Nagelkerke’s pseudo R^2^ and the Hosmer-Lemeshow test.

**Table S1.** Multivariate Logistic Regression Analysis of LTF Association With Advanced TNM Stage Adjusted for Clinical Confounders

| **Variable** | **β (SE)** | **OR (95% CI)** | **P Value** | |
| --- | --- | --- | --- | --- |
| LTF-1 | 2.357(1.035) | 10.55(1.596 - 105.0) | 0.0227* | |
| Gender(M 1,F 0) | -0.9467(1.487) | 0.3880(0.01842 -7.840) | 0.5245 | |
| Age（<60 1,60-80 2.>80 3） | 2.333(1.374) | 10.31(0.9115 -244.6) | 0.0896 | |
| smoking | 0.8270(1.488) | 2.286(0.1128 - 49.51) | 0.5785 | |
| T | 1.735(0.8860) | 5.669(1.254 - 46.59) | 0.0502 | |
| N |  |  |  | |
| M |  |  |  | |
|  | | | |  |
|  | | | |  |
|  | | | |  |
|  | | | |  |
|  | | | |  |
