# Supplementary material for: Lactotransferrin upregulation affects the pathological changes of non-small cell lung cancer by regulating ferroptosis
Source: PeerJ. 2026 Feb 27;14:e20866. doi: 10.7717/peerj.20866 (PMC12951881; doi:10.7717/peerj.20866)
Supplement: Supplemental Information 15 [file peerj-14-20866-s015.docx]

**Supplementary Materials Template for Lentiviral Vector Details**

**1. Lentiviral Vector Construction**

|  | **Backbone Vector** | **Target Sequences** | **Packaging System** |
| --- | --- | --- | --- |
| LTF-shRNA1 | PLVX-shRNA2-puro | GGATCAGAAAGATCTGCTGTTCAACTCGAGTTGAACAGCAGATCTTTCTTTTTTTAATTC | PKG:PKGX and pMD2.G for lentivirus production. |
| LTF-shRNA2 | PLVX-shRNA2-puro | GGATCGATGAATATTTCAGTCAAACTCGAGTTTGACTGAAATATTCATCTTTTTTAATTC |  |
| LTF-shRNA3 | PLVX-shRNA2-puro | GGATCGGAACTCTGTGAAAGGCAACTCGAGTTGCCTTTCACAGAGTTCCTTTTTTAATTC |  |
| LTF-oeRNA | pLV-CMV-MCS-EF1-ZsGreen1-T2A-Puro | CTCGAGATGAAACTTGTCTTCCTCGTCCTGCTGTTCCTCGGGGCCCTCGGACTGTGTCTGGCTGGCCGTAGGAGGAGTGTTCAGTGGTGCGCCGTATCCCAACCCGAGGCCACAAAATGCTTCCAATGGCAAAGGAATATGAGAAAAGTGCGTGGCCCTCCTGTCAGCTGCATAAAGAGAGACTCCCCCATCCAGTGTATCCAGGCCATTGCGGAAAACAGGGCCGATGCTGTGACCCTTGATGGTGGTTTCATATACGAGGCAGGCCTGGCCCCCTACAAACTGCGACCTGTAGCGGCGGAAGTCTACGGGACCGAAAGACAGCCACGAACTCACTATTATGCCGTGGCTGTGGTGAAGAAGGGCGGCAGCTTTCAGCTGAACGAACTGCAAGGTCTGAAGTCCTGCCACACAGGCCTTCGCAGGACCGCTGGATGGAATGTCCCTATAGGGACACTTCGTCCATTCTTGAATTGGACGGGTCCACCTGAGCCCATTGAGGCAGCTGTGGCCAGGTTCTTCTCAGCCAGCTGTGTTCCCGGTGCAGATAAAGGACAGTTCCCCAACCTGTGTCGCCTGTGTGCGGGGACAGGGGAAAACAAATGTGCCTTCTCCTCCCAGGAACCGTACTTCAGCTACTCTGGTGCCTTCAAGTGTCTGAGAGACGGGGCTGGAGACGTGGCTTTTATCAGAGAGAGCACAGTGTTTGAGGACCTGTCAGACGAGGCTGAAAGGGACGAGTATGAGTTACTCTGCCCAGACAACACTCGGAAGCCAGTGGACAAGTTCAAAGACTGCCATCTGGCCCGGGTCCCTTCTCATGCCGTTGTGGCACGAAGTGTGAATGGCAAGGAGGATGCCATCTGGAATCTTCTCCGCCAGGCACAGGAAAAGTTTGGAAAGGACAAGTCACCGAAATTCCAGCTCTTTGGCTCCCCTAGTGGGCAGAAAGATCTGCTGTTCAAGGACTCTGCCATTGGGTTTTCGAGGGTGCCCCCGAGGATAGATTCTGGGCTGTACCTTGGCTCCGGCTACTTCACTGCCATCCAGAACTTGAGGAAAAGTGAGGAGGAAGTGGCTGCCCGGCGTGCGCGGGTCGTGTGGTGTGCGGTGGGCGAGCAGGAGCTGCGCAAGTGTAACCAGTGGAGTGGCTTGAGCGAAGGCAGCGTGACCTGCTCCTCGGCCTCCACCACAGAGGACTGCATCGCCCTGGTGCTGAAAGGAGAAGCTGATGCCATGAGTTTGGATGGAGGATATGTGTACACTGCAGGCAAATGTGGTTTGGTGCCTGTCCTGGCAGAGAACTACAAATCCCAACAAAGCAGTGACCCTGATCCTAACTGTGTGGATAGACCTGTGGAAGGATATCTTGCTGTGGCGGTGGTTAGGAGATCAGACACTAGCCTTACCTGGAACTCTGTGAAAGGCAAGAAGTCCTGCCACACCGCCGTGGACAGGACTGCAGGCTGGAATATCCCCATGGGCCTGCTCTTCAACCAGACGGGCTCCTGCAAATTTGATGAATATTTCAGTCAAAGCTGTGCCCCTGGGTCTGACCCGAGATCTAATCTCTGTGCTCTGTGTATTGGCGACGAGCAGGGTGAGAATAAGTGCGTGCCCAACAGCAACGAGAGATACTACGGCTACACTGGGGCTTTCCGGTGCCTGGCTGAGAATGCTGGAGACGTTGCATTTGTGAAAGATGTCACTGTCTTGCAGAACACTGATGGAAATAACAATGAGGCATGGGCTAAGGATTTGAAGCTGGCAGACTTTGCGCTGCTGTGCCTCGATGGCAAACGGAAGCCTGTGACTGAGGCTAGAAGCTGCCATCTTGCCATGGCCCCGAATCATGCCGTGGTGTCTCGGATGGATAAGGTGGAACGCCTGAAACAGGTGTTGCTCCACCAACAGGCTAAATTTGGGAGAAATGGATCTGACTGCCCGGACAAGTTTTGCTTATTCCAGTCTGAAACCAAAAACCTTCTGTTCAATGACAACACTGAGTGTCTGGCCAGACTCCATGGCAAAACAACATATGAAAAATATTTGGGACCACAGTATGTCGCAGGCATTACTAATCTGAAAAAGTGCTCAACCTCCCCCCTCCTGGAAGCCTGTGAATTCCTCAGGAAGTAAGGATCC |  |

**2. Multiplicity of Infection (MOI) Optimization**

MOI = 15 of A549 cells was determined by pilot transduction with GFP reporter virus (Figure S4A). MOI = 12 of H1299 cells was determined by pilot transduction with GFP reporter virus (Figure S4B).

LV-Enhance (6 μg/mL) enhanced transduction efficiency.

**3. transduction efficiency.**

The lentiviral transfection efficiencies of A549 cells and H1299 cells are shown in Figures S4C and S4D.

**4.Positive controls of ROS, Fe2+ and TEM imaging**

1.ROS Probe (DCFH-DA/C11-BODIPY) Assay:

Positive Control: Beas-2B Cells treated with 100 μM tert-butyl hydroperoxide (TBHP) for 2 h (known ROS inducer) were used alongside experimental groups (Supplementary Figure S3A). This treatment induced significantly increase in DCF fluorescence vs. untreated cells (p<0.001), validating probe sensitivity.

2. Fe²⁺ Probe (FerroOrange/RPA) Staining:

Positive Control: Cells incubated with 10 μM Erastin for 24 showed a significant FerroOrange signal boost (Supplementary Figure S3B). This confirmed Fe²⁺-specific detection under our experimental conditions.

3. TEM Imaging:

Positive Control: Cells treated with 10 μM Erastin for 24 h (classic ferroptosis inducer) exhibited characteristic mitochondrial shrinkage and increased membrane density, distinct from apoptosis/necrosis (Supplementary Figure S3C). These features matched prior report.
